# Unravelling the nature of magneto-electric coupling in room temperature multiferroic particulate (PbFe_0.5_Nb_0.5_O_3_)–(Co_0.6_Zn_0.4_Fe_1.7_Mn_0.3_O_4_) composites

**DOI:** 10.1038/s41598-021-82399-7

**Published:** 2021-02-04

**Authors:** Krishnamayee Bhoi, H. S. Mohanty, Ravikant, Md. F. Abdullah, Dhiren K. Pradhan, S. Narendra Babu, A. K. Singh, P. N. Vishwakarma, A. Kumar, R. Thomas, Dillip K. Pradhan

**Affiliations:** 1 Department of Physics and Astronomy, National Institute of Technology, Rourkela, Odisha 769008 India; 2 CSIR National Physical Laboratory, Dr. K. S. Krishnan Marg, New Delhi, 110012 India; 3 Extreme Materials Initiative, Geophysical Laboratory, Carnegie Institution for Science, Washington, DC 20015 USA; 4 Materials Research Laboratory, Department of Physics, Osmania University, Hyderabad, 500007 India; 5 Division of Research and Development, Lovely Professional University, Jalandhar-Delhi G.T. Road, Phagwara, Punjab 144411 India; 6 School of Chemical Engineering and Physical Sciences, Lovely Professional University, Jalandhar-Delhi G.T. Road, Phagwara, Punjab 144411 India; 7 Present Address: Department of Basic Science and Humanities, GIET University, Gunupur, Odisha 765022 India; 8 Present Address: Department of Physics, Government College Sailana, Vikram University, Ujjain, 457550 India

**Keywords:** Materials science, Physics

## Abstract

Multiferroic composites are promising candidates for magnetic field sensors, next-generation low power memory and spintronic devices, as they exhibit much higher magnetoelectric (ME) coupling and coupled ordering parameters compared to the single-phase multiferroics. Hence, the 3-0 type particulate multiferroic composites having general formula (1 − Φ)[PbFe_0.5_Nb_0.5_O_3_]-Φ[Co_0.6_Zn_0.4_Fe_1.7_Mn_0.3_O_4_] (Φ = 0.0, 0.05, 0.1, 0.2, 0.3, 0.4, 0.5, 1.0, (1 − Φ) PFN-ΦCZFMO) were prepared using a hybrid synthesis technique. Preliminary structural and microstructural analysis were carried out using XRD and FESEM techniques, which suggest the formation of 3-0 type particulate composite without the presence of any impurity phases. The multiferroic behaviour of the composites is studied with polarization versus electric field (P-E) and magnetization versus magnetic field (M-H) characteristics at room temperature. The nature of ME coupling was investigated elaborately by employing the Landau free energy equation along with the magneto-capacitance measurement. This investigation suggests the existence of biquadratic nature of ME coupling (P^2^M^2^). The magneto-electric coupling measurement also suggests that strain mediated domain coupling between the ferroelectric and magnetic ordering is responsible for the magneto-electric behaviour. The obtained value of direct ME coefficient 26.78 mV/cm-Oe for Φ = 0.3, found to be higher than the well-known single-phase materials and polycrystalline composites.

## Introduction

Multiferroics materials that simultaneously exhibit two or more primary ferroic ordering like ferroelectric (FE), ferromagnetic (FM), and ferroelastic, are of immense technological importance^1^. The underlying physics in these classes of materials and their utilization for the development of multifunctional devices have drawn tremendous research interest among the scientific community^2^. The term “Magnetoelectric” is associated with the coupling between ferroelectric and magnetic order parameters. This allows the tuning and switching of electrical polarization (P) with an applied magnetic field (H) and is known as Direct ME Effect (DME). In case of Converse ME Effect, (CME), the magnetization (M) can be tuned and switched with an external electric field (E). In DME, “P” and “H” are connected by the relation ΔP = α_H_ΔH, whereas “M” and “E” are related by µ_0_ΔM = α_E_ΔE in the case of CME. Here, µ_0_ is the vacuum permeability, and α_H_ or α_E_ corresponds to ME coupling coefficients, which quantifies the ME response of the material. The magnitude of α_H_ or α_E_ represents the coupling strength between electric and magnetic order parameters^3^. Owing to the mutual exclusiveness and chemical incompatibility for the occurrence of ferroelectricity and magnetism, single-phase multiferroics with strong magneto-electric coupling are very rare in nature. Generally, multiferroic materials are available in the form of single-phase and composite structures^2^. Single-phase magneto-electric multiferroic systems possess at least two primary ferroic orders in a single system whereas composites refer to multiphase materials with multiferroic properties arising out of the interactions of respective phases^4^. Most of the available single-phase room temperature (RT) multiferroic materials show very weak ME coupling due to the independent ordering of ferroelectricity (FE) and magnetism. On the other hand, they show good ME coupling at cryogenic temperatures (magnetic/FE transitions are below RT)^2,5^. Strong ME coupling between “M” and “P” around and above RT is the essential requirement for the realization of multifunctional devices^4,6^. So, there is a scientific urge to design the composite magneto-electric materials by combining different ferroic phases so that substantial ME coupling occurs between the respective phases at RT or above.

Composites can be formed by combining two or more phases in different connectivity to achieve an excellent ME effect above RT^6^. Out of two types of composites (bulk and layered), bulk composites have the advantages over layered composite in respect of mechanical strength, easy control of different parameters like magnetic, electric, and ME coefficient with proper selection of individual phases^7^. But in the case of bulk composite, the experimental value of α_E_ is somehow less than the expected value. The possible reasons behind such discrepancies in ferrite based composites are (i) due to the presence of low resistive ferrite phase, the electrical shunting between the piezoelectric phases leading to loss of piezoelectric properties, (ii) the unavoidable presence of Fe^2+^ in the ferrite phase increases the leakage current of the material (iii) formation of micro-cracks due to difference in the thermal expansion coefficients of the constituent phases and (iv) porosity and undesired phases formation^7^._._ The ME effect in composites results extrinsically through the mediation of strain, charge carrier, and spin-exchange. Among these phenomena, ME effect through strain mediation has been studied widely^3,8^. However, the other two phenomena i.e., mediation through charge carrier and spin exchange, are not well understood and need to be studied^3,8^. The ME effect via strain mediation in composite results from the interaction between ferrite (magnetostrictive) phase and ferroelectric (piezoelectric) phases^9,10^. The strain mediated composites can be prepared by combining piezomagnetic/magnetostrictive and piezoelectric phases. To establish a stronger ME effect in such composites, a ferrite phase with a larger magnetostrictive coefficient and a ferroelectric phase with a larger piezoelectric coefficient are essential^7^. Based on this concept, research work are mainly concentrated on ferroelectric- ferrite composites such as : Pb(Zr_0.53_Ti_0.47_)O_3_-CoFe_2_O_4_^11^, Pb(Zr_0.52_Ti_0.48_)O_3_-NiFe_2_O_4_^12^, (1 − *x*)BiFe_0.5_Cr_0.5_O_3_-*x*NiFe_2_O_4_^13^, BaTiO_3_-MgFe_2_O_4_^14^, Pb(Zr_0.53_Ti_0.47_)O_3_-Ni_0.5_Zn_0.5_Fe_2_O_4_^15^, 0.9BaTiO_3_-0.1Ni_*x*_Zn_1-*x*_Fe_2_O_4_^16^, (1 − *x*)Pb(Fe_0.5_Nb_0.5_)O_3_-*x* Ni_0.65_Zn_0.35_Fe_2_O_4_^17^, Pb(Fe_0.5_Nb_0.5_)O_3_-Co_0.65_Zn_0.35_Fe_2_O_4_^5^, (1 − *x*) GaFeO_3_-*x*Co_0.5_Zn_0.5_Fe_2_O_4_^18^. A detailed literature survey on multiferroic composites demonstrates that most of the works are based on the synthesis and studies on the magnetic, ferroelectric, dielectric, magneto-dielectric (MD) and ME properties with the emphasis on the enhancement of ME coupling. Very few reports are available which mention the exact nature of coupling (direct or indirect) in these composites at RT. The magneto-electric property (functionality) of the composite is product property relation which is not observed in either of the ferrite (magnetostrictive) phase and ferroelectric (piezoelectric) phase. Although, it has been reported that, the appearance of strain mediated ME coupling between these two constituent phases (i.e., magnetostrictive and piezoelectric) of the composite might be originating at the interface, but no systematic reports are available on this mechanism^10^. These findings motived us to study the nature and origin of magneto-electric coupling in particulate composites.

The key parameters such as dielectric constant, piezoelectric constant, mechanical quality factor, magnetostrictive coefficient, Curie, and Neel temperature should be examined properly before the selection of the individual phases for the composite formation^8^. PbFe_0.5_Nb_0.5_O_3_ (PFN) is a single-phase multiferroics compound with high dielectric constant (3000 at 1 kHz), low dielectric loss (tanδ = 0.01 at 1 kHz), high piezoelectric coefficient (d_33_ = 145 pC/N), good FE behaviour, weak magnetic properties with the existence of ME effect at low temperature^19^. It undergoes a ferroelectric-paraelectric transition (T_C_) around 383 K, antiferromagnetic-paramagnetic transition (T_N_) around 122–145 K, and a weak FM below 10 K^19^. PFN also shows weak ferromagnetic behaviour around 300 K or above due to spin clustering and canted antiferromagnetic ordering of spins^20^. On the other hand, among the oxide-based magnetostrictive materials, the highest value of λ (absolute magnetostriction coefficient) = − 110 × 10^–6^ with M_S_ (saturation magnetization) = 81 emu/g is reported for CoFe_2_O_4_ (CFO)^21^. However, it exhibits the disadvantages such as high coercivity and large anisotropy. Substitution of small amount of Mn at the Fe-sites of CFO, i.e., CoFe_1.7_Mn_0.3_O_4_ (CFMO), reduces the anisotropy constant and coercivity with $\frac{d\lambda }{{dH}} \approx 2.5 \times 10^{ - 9} \text{m}/\text{A}$ at low field. On the other hand, substitution of Zn for Co at the tetrahedral site of CFO also reduces the magnetic anisotropy. The optimized composition Co_0.6_Zn_0.4_Fe_1.7_Mn_0.3_O_4_ (CZFMO) showed maximum magnetostriction coefficient, high resistivity (≈ 10^8^ Ω cm), and comparable magnetization^21^. Therefore, improved values of magneto-electric coefficients can be achieved on CZFMO based composites^21–24^. In this report, our main goal is to investigate the nature and the origin of strain mediated ME coupling in PbFe_0.5_Nb_0.5_O_3_- Co_0.6_Zn_0.4_Fe_1.7_Mn_0.3_O_4_ (PFN-CZFMO) composite systems.

## Experimental details

### Synthesis procedure

Besides the selection of material, the synthesis technique adopted to prepare the composites play a great role in enhancing the multiferroic behaviour. So during the synthesis of composites, three essential issues such as (i) chemical reaction between the constituent phases, (ii) inhomogeneous dispersion of ferrite phase in the matrix, and (iii) the mechanical defects such as pore trapped at the interface of the constituent phases are reported. All these factors deteriorate the functional properties of the composites. Powder-in-sol precursor hybrid processing route is known to be one of the good synthesis techniques to address the aforementioned problems^15,25^. This processing route combines the advantageous properties of solid-state reaction such as high crystallinity, high performance along with fine grain and low sintering temperature of the sol–gel method^15,25^. The multiferroic PFN was prepared using solid-state reaction route, whereas ferrite phase CZFMO was prepared using the sol–gel method. The different weight percentages of PFN and CZFMO were prepared using solid-state reaction route to prepare the desired composites. Multiferroic ceramic composites of (1 − Φ) PbFe_0.5_Nb_0.5_O_3_-ΦCo_0.6_Zn_0.4_Fe_1.7_Mn_0.3_O_4_ (Φ = 0.0, 0.05, 0.1, 0.2, 0.3, 0.4, 0.5, 1.0) were synthesized using this hybrid synthesis technique.

For the synthesis of PFN, high-grade chemicals of PbO, Fe_2_O_3,_ and Nb_2_O_5_ were taken as the precursors of Pb, Fe, Nb, respectively. Initially, these ingredients were weighted in stoichiometric proportion; however, 7% extra PbO (optimized) was taken in order to compensate Pb-loss during the synthesis process. These precursors were mixed in an agate mortar for 1 h in the ambient condition and then grinded for 3 h in acetone medium. The calcination process was carried out at 920 °C for 6 h. The process of calcination and grinding was continued until the formation of single-phase material and finally sintered at 980 °C for 6 h. A single phase Co_0.6_Zn_0.4_Fe_1.7_Mn_0.3_O_4_ was synthesized using the sol–gel method. For this, (CH_3_COO)_2_Co·4H_2_O, Fe(NO_3_)_3_·9H_2_O, Zn(NO_3_)_2_·6H_2_O, Mn(CH_3_COO)_2_·4H_2_O were taken as precursors. (CH_3_COO)_2_Co·4H_2_O was dissolved in distilled water followed by the addition of Fe(NO_3_)_3_·9H_2_O, Zn(NO_3_)_2_·6H_2_O, and Mn(CH_3_COO)_2_·4H_2_O in the desired molar ratio of Co, Zn, Fe and Mn. After that, citric acid (C_6_H_8_O_7_) was added to the solution, which acts as the fuel to drive the combustion process. Here, the ratio of citric acid to the precursors is fixed at 2:1. The pH of the solution was maintained at 7 by the addition of ammonium hydroxide. The clear solution was then constantly heated and mixed properly under magnetic stirring to get the gel. The gel spontaneously converted to combustion residue by drying it at 250 °C. The obtained residue was then grinded and calcined at an optimized temperature of 700 °C for 2 h and sintered at 900 °C for 2 h. Now for the synthesis of multiferroic composites of (1 − Φ) PFN-Φ CZFMO, the calcined powders of individual phases were taken in different weight percentages. These powders were mixed in the mortar pestle for 30 min in ambient condition and then 1 h in acetone medium. The 3 wt% of polyvinyl alcohol (PVA) solution was added to it to fabricate the pellets using a uni-axial hydraulic press. These pellets are then subjected for sintering (evaporation of PVA was performed first) at 980 °C for 6 h to improve the densification of ceramic composites.

### Characterization techniques

In order to check the formation of individual phases and the formation of multiferroic composites, the X-ray diffraction (XRD) technique was performed using D2-PHASER (Bruker) X-ray diffractometer with Cu Kα_1_ radiation (λ = 1.5405 Å). The RT XRD data was taken in a wide range of Bragg’s angle 20°–80° at a scanning rate of 2°/min with 0.02° step size. The surface morphology, grain distributions of both the phases in ceramic composites were studied using Field Emission Scanning Electron Microscopy (FESEM) technique using NOVA NanoSEM 450 at RT. The high-resolution transmission electron micrograph (HRTEM), and selective area electron diffraction (SAED) patterns of CZFMO phase were recorded using Field Electron and Ion (FEI), Tecnai G2 TF30-ST transmission electron microscope (TEM).The magnetic hysteresis measurement was performed using a vibrating sample magnetometer (Lakeshore USA 7404) in the field range of − 1.5 T to 1.5 T at RT. Variation of ferroelectric polarization with the electric field of the prepared materials was carried out at a constant frequency of 100 Hz using Radiant ferroelectric tracer. The piezoelectric coefficient (d_33_) values are measured with the help of d_33_ piezometer APC International Ltd. Before piezoelectric and ferroelectric measurements, samples are poled at 20 kV/cm. The magneto-dielectric (MD) properties were measured using a high precession impedance analyzer (Wayne Kerr 6500B) over the frequency range 100 Hz to 1 MHz in the presence of various static magnetic fields at RT. The magnetic fields needed for the MD measurement was obtained from an electromagnet (GMW-5403) attached with a bipolar dc supply. The magnetoelectric (ME) measurements of the composite samples were carried out at room temperature by the dynamic method using an indigenous ME set up^26^. For the ME measurement, the samples were kept between the pole pieces of a DC electromagnet, which can generate the DC magnetic field up to 5 kOe. The AC Helmholtz coils each of 130 turns were mounted on the DC magnet pole pieces. The coils are excited with a low-amplitude high-frequency (1.008 kHz) signal, using the internal oscillator of the dual phase lock-in-amplifier (Make: Stanford Research Systems, Model SR530). Since the signal current was very small to drive the coils, a power amplifier (Make: Ahuja, Model: TZA 4000) was used to amplify the current. The AC magnetic field thus generated was calculated knowing the current carried by the coils using a Keithley 196 DMM. The whole setup was properly shielded to avoid stray pickups. The magnetoelectric voltage coefficient is determined by measuring the induced voltage generated across the sample when an AC magnetic field and a DC bias are applied to it. In the dynamic method, ME output was measured at a constant AC magnetic field of 1 Oe (with frequency *f* = 1.008 kHz) superimposed on a varying DC field in the range of 0 – 5 kOe. The ME output in these composites was recorded as a function of DC bias field at fixed value of AC field of 1 Oe (*f* = 1008 kHz) using a lock-in-amplifier (Make: Stanford Research Systems, Model SR530). The direction of both the applied AC and DC magnetic fields were along the thickness of the samples. The magnetoelectric output (*E*) = induced voltage (V)/thickness of the sample (d), units are mV/cm. Then the magnetoelectric voltage coefficient is equal to (d*E*/d*H)*, having unit mV/cm-Oe where, *E* is the magnetoelectric output and *H* is the applied AC magnetic field.

## Results discussion

### Structural and morphological properties

#### X-ray diffraction

**Figure 1:**
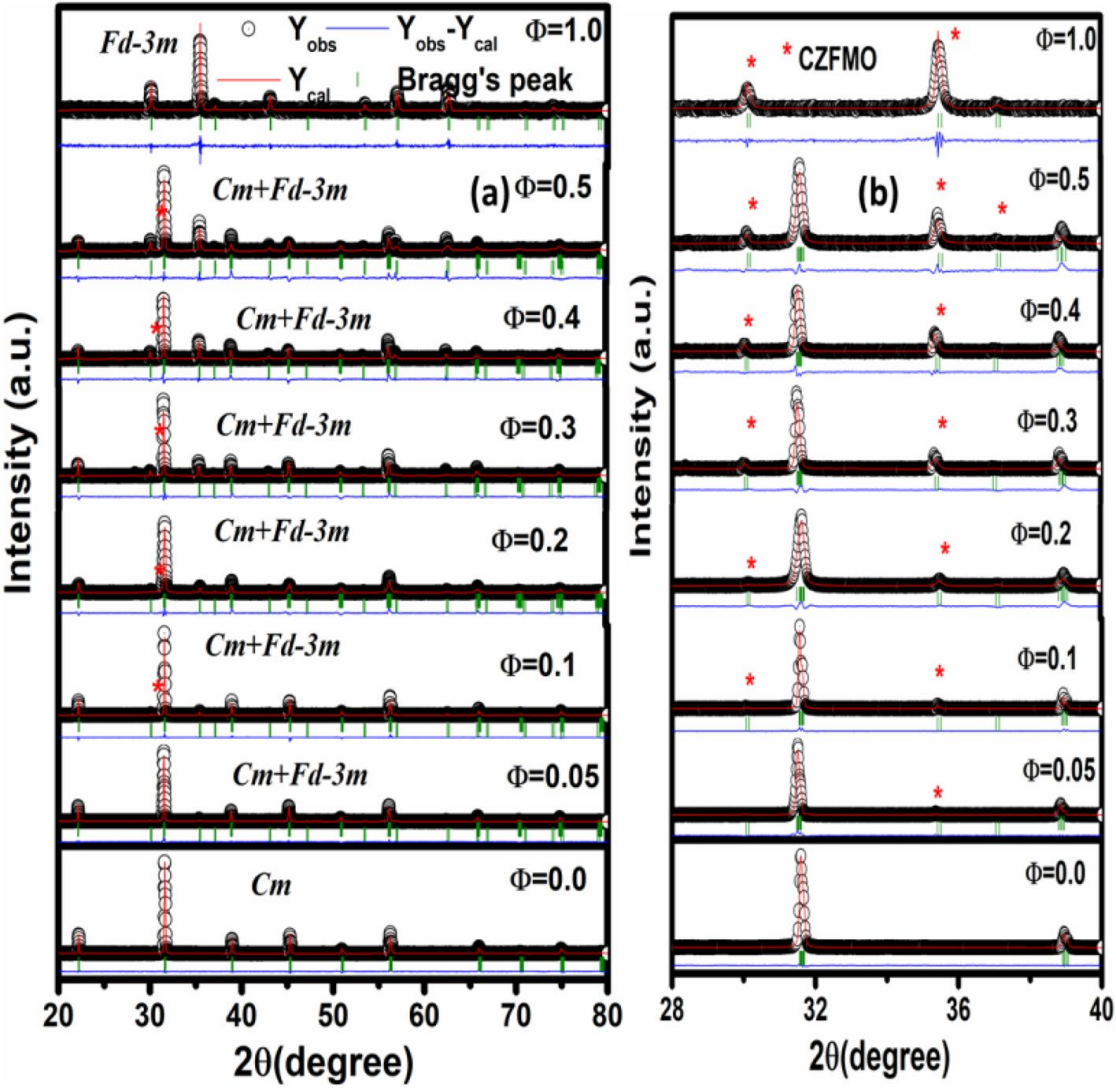
(**a**) The Rietveld refinement result of XRD patterns of (1 − Φ) PbFe_0.5_Nb_0.5_O_3_-ΦCo_0.6_Zn_0.4_Fe_1.7_Mn_0.3_O_4_ ((1 − Φ) PFN-Φ CZFMO) (Φ = 0.0, 0.05, 0.1, 0.2, 0.3, 0.4, 0.5, 1.0) composites at RT. (**b**) Enlarged view of the XRD patterns in the 2θ range 28°–40° to show the formation of composite with the appearance of reflection for both the constituent phases.

Figure 1 depicts the X-ray diffraction patterns of (1 − Φ) PFN-Φ CZFMO (Φ = 0.0, 0.05, 0.1, 0.2, 0.3, 0.4, 0.5, 1.0) composites recorded at RT. The XRD patterns of Φ = 0.0 (i.e., PFN) show the formation of highly crystalline material with single-phase perovskite structure, which is well-matched with the literature^5,17^. The peaks of Φ = 1.0 (i.e., CZFMO) indicate the appearance of high purity and good crystallinity with spinel structure single-phase CZFMO. The XRD pattern of CZFMO is in accordance with the earlier report^24^. In the composite (Φ = 0.05, 0.1, 0.2, 0.3, 0.4, 0.5), the diffraction patterns clearly show the existence of perovskite PFN phase and spinel phase of CZFMO without a trace of any other secondary phases. So during the synthesis process of the composites, no chemical reaction occurred between the individual phases and thus confirms the formation of composite with the simultaneous presence of parental PFN and CZFMO phase using a hybrid synthesis technique. In order to understand the evolution of the composite nature with increase in the CZFMO concentrations, the XRD patterns in the Bragg’s angle ranging 28°–40° is shown separately in Fig. 1b. It has been observed that with the increase in CZFMO content, the peak intensity of the ferrite phase increases while the peak intensity of the PFN phase decreases.

Further, the Rietveld refinement technique was adopted to (i) confirm composite phase formation, (ii) quantification of individual phase fraction using Fullprof package^27^. The multiferroic perovskite PFN compound crystallizes in monoclinic crystal structure with space group *Cm* whereas magnetostrictive phase CZFMO crystallizes in cubic structure with space group *Fd-3m*^5,28^. So, during the refinement process, *Cm* space group was used to fit the XRD pattern of PFN, and for CZFMO, *Fd-3m* space group was chosen. The in-between compositions were refined by taking dual-phase i.e., monoclinic and cubic phase (*Cm* + *Fd-3m*), which also gives a good fit between the experimental and simulated data. The experimental and theoretical data has been found to match well for all values of Φ (with goodness of fit parameters χ^2^ in the range 1.37 ≤ χ^2^ ≤ 4.55). This enabled us to confirm the proposed presence of dual-phase and hence confirm the formation of the composite. All the refined parameters are listed in Table 1. It has been observed that the unit cell volume of the PFN phases decreases, whereas for the CZFMO phase, the unit cell volume increases with increase in the CZFMO concentrations. The Rietveld refinement result also shows the presence of the phase fraction of the different phases, as shown in Table 1. The observed phase fraction of both the phases well matched with the proposed value.

**Table 1:** Crystal structure parameters (obtained from Rietveld refinement analysis) and the micro-strain and average crystallite size of PFN and CZFMO phase (obtained from W–H plot) of (1 − Φ) PFN-Φ CZFMO (Φ = 0.0, 0.05, 0.1, 0.2, 0.3, 0.4, 0.5, 1.0) composites.

Sample name	Space group	a (Å)	b (Å)	C (Å)	V (Å^3^)	Phase fraction (%)	Crystallite size (nm)	Micro-strain (× 10^–3^)	χ^2^
Φ = 0.0 (PFN)	*Cm*	5.6746 (1)	5.6680 (1)	4.0175 (1)	129.21 (7)	100			2.84
Φ = 0.1	*Cm* + *Fd-3m*	5.6735 (2)	5.6650 (1)	4.0152 (9)	129.05 (2)	89.8	58.5	1.58	3.74
		8.3982 (6)	8.3982 (6)	8.3982 (6)	592.32 (3)	10.2	49.9	1.46	
Φ = 0.2	*Cm* + *Fd-3m*	5.6730 (1)	5.6640 (1)	4.0148 (2)	129.03 (2)	83.1	57.5	1.59	4.47
		8.3995 (9)	8.3995 (9)	8.3995 (9)	592.59 (3)	16.9	50.1	1.51	
Φ = 0.3	*Cm* + *Fd-3m*	5.6724 (4)	5.6636 (4)	4.0140 (7)	128.95 (1)	66.8	56.0	1.61	4.55
		8.4026 (1)	8.4026 (1)	8.4026 (1)	593.25 (2)	33.2	51.9	1.58	
Φ = 0.4	*Cm* + *Fd-3m*	5.6720 (5)	5.6631 (5)	4.0137 (2)	128.92 (3)	56.3	52.6	1.35	3.74
		8.4031 (2)	8.4031 (2)	8.4031 (2)	593.37 (3)	43.7	50.0	1.56	
Φ = 0.5	*Cm* + *Fd-3m*	5.6712 (9)	5.6624 (9)	4.0131 (2)	128.87 (3)	49.1	51.6	1.25	2.99
		8.4042 (9)	8.4042 (9)	8.4042 (9)	593.59 (1)	50.9	50.1	1.50	
Φ = 1.0 CZFMO	*Fd-3m*	8.3957 (1)	8.3957 (1)	8.3957 (1)	591.80 (2)	100	31.8	1.67	1.37

The Williamson–Hall (W–H) method is used to calculate the average crystallite size, microstrain of PFN and CZFMO phase. The equation used for W–H method can be expressed as^30^

1 $$ \beta \cos \theta = 4\varepsilon \sin \theta + \frac{K\lambda }{D} $$

where *β* = fullwidth half maxima (FWHM), *ε* = lattice strain, *K* = shape parameter, *D* = average crystallite size and *λ* = wavelength of the XRD. The W–H plots for PFN and CZFMO phases are plotted by calculating the FWHM of the individual phase from the fitting of XRD patterns of the composites. The variation of βcosθ vs. 4sinθ for PFN and CZFMO phases have been plotted for the composition Ф = 0.3 (as representative) is shown in supplementary materials Fig. S1a,b respectively. Similar behaviours have been observed for all other compositions. The microstrain can be calculated from the slope and crystallite size was calculated from the Y-axis intercept. The calculated values of lattice strain and average crystallite size is listed in Table 1. From Table 1 it has been observed that the micro-strain for both the PFN and CZFMO phases increases with the increase in CZFMO concentrations and the maximum values of micro-strain is observed for Φ = 0.3. Above this concentration, the micro-strain value decreases. The maximum value of micro-strain for Φ = 0.3, may be due to the evolution of CZFMO phase with respect to PFN as observed in the FESEM study (discussed later). The average crystallite size of PFN decreases with increase in CZFMO content and reverse trend has been observed for CZFMO phase with increase in Φ. This behaviour is similar to the variation of the grain size as obtained from the FESEM analysis, which is discussed in the later section.

#### Transmission electron and field emission scanning microscopy

Figure S2a shows the TEM image of CZFMO nanoparticles. The TEM image suggests the average size of the particles range between 40 and 50 nm. The HRTEM image and SAED pattern of CZFMO are shown in Fig. S2b,c, respectively. The clear fringes of HRTEM image indicate the formation of CZFMO with good quality of crystallinity. The inter-planar spacing, “d”-value calculated from the HRTEM image corresponds to (311) plane (d = 0.250 nm) of the spinel structure of CZFMO. This result is in good agreement with the XRD data. The SAED image conveys the polycrystalline nature of CZFMO. The concentric diffraction rings from inside to outside are assigned to (111), (220), (311), (222), (400), (422), (511), (420), (553) crystal planes corresponds to the cubic CZFMO, also matched with the XRD data. The HAADF-STEM (High-angle Annular Dark Field Scanning TEM) micrograph of CZFMO with the elemental mapping is illustrated in Fig. S2d. This micrograph shows the presence of Co, Zn, Fe, Mn, and O (indicated with their respective colours) in Fig. S2d and homogeneous distribution of these elements throughout the sample microstructure. This study suggests the formation of good quality CZFMO nanoparticles.

**Figure 2:**
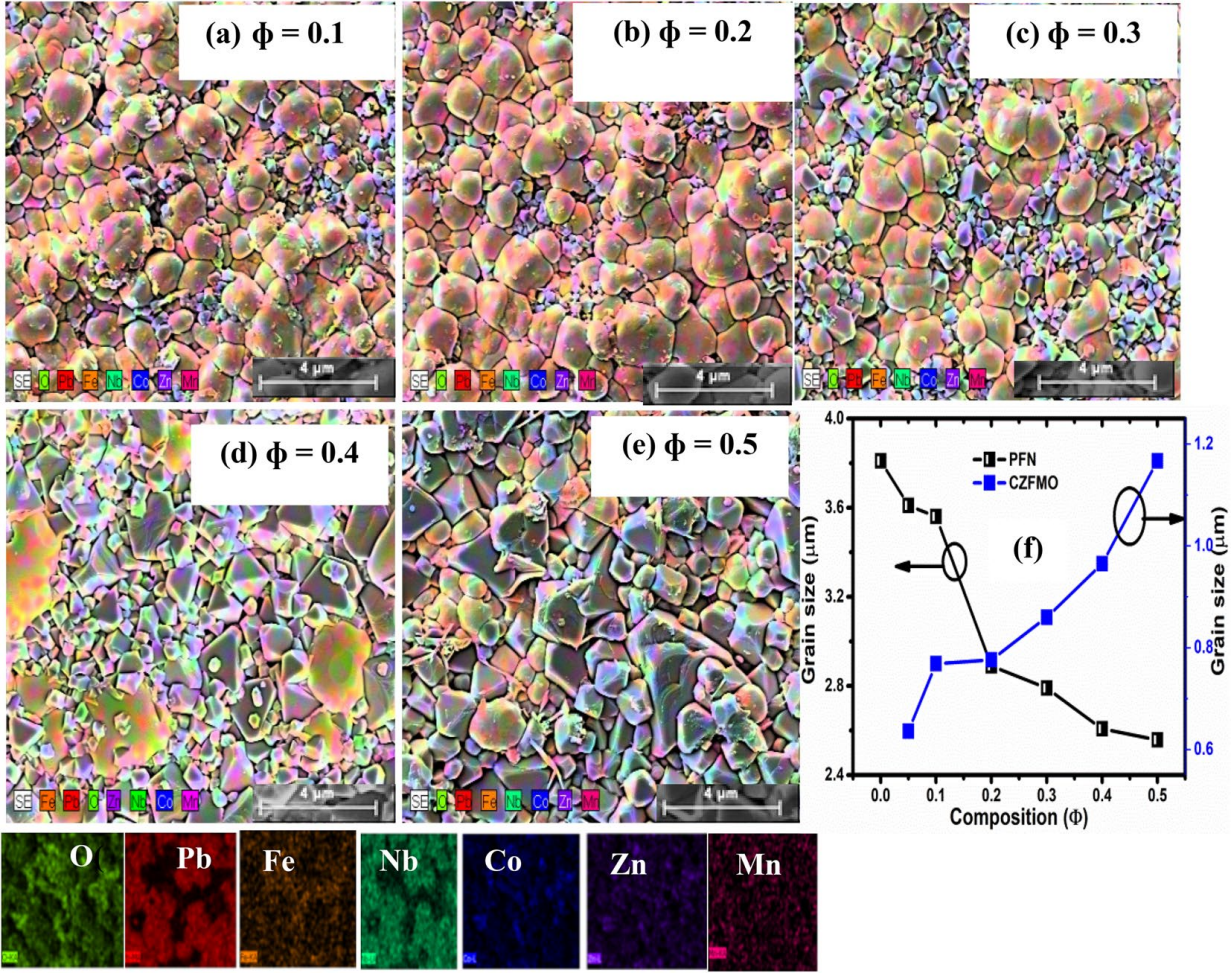
The FESEM micrographs of (**a**) Φ = 0.1 (**b**) Φ = 0.2 (**c**) Φ = 0.3 (**d**) Φ = 0.4 and (**e**) Φ = 0.5 of (1 − Φ) PbFe_0.5_Nb_0.5_O_3_-ΦCo_0.6_Zn_0.4_Fe_1.7_Mn_0.3_O_4_ composite. (**f**) Variation of average grain sizes of PFN and CZFMO with Φ.

The FESEM micrograph of pure PFN (shown in Supplementary Materials Fig. S3a) shows polyhedral grains of different sizes that are homogeneously distributed throughout the sample microstructure. The microstructure is mostly pore-free, and the grain growth process appears to be completed during the sintering process. The average grain size of PFN was calculated using Image J software and found to be around 3–4 µm. In order to investigate the existence of all cationic elements in PFN, EDS analysis has been carried out. The presence of Pb, Fe, Nb and O elements is ascertained from the EDS spectrum of PFN (Supplementary Materials Fig. S3b). The FESEM micrographs with elemental mapping of (1 − Φ) PFN-Φ CZFMO composites for Φ = 0.1, 0.2, 0.3, 0.4, and 0.5 are shown in Fig. 2a–e. The corresponding colour for the presence of elements i.e., Pb, Fe, Nb, Co, Zn, Mn, and O are shown in the bottom of Fig. 2a–e. The FESEM micrographs suggest the formation of pore-free and dense grains which are separated by well-defined grain boundaries. So, there is a formation of high-quality materials with the completion of the grain growth process. The presence of two different types of grains, polyhedral larger grains of PFN phase and random size grains of CZFMO phase, are also observed in these micrographs. The polyhedral larger grains correspond to matrix, whereas random shaped smaller grains correspond to the island. This can be verified from the elemental mapping of the micrograph. In Fig. 2a–e, in the larger grains, the content of Pb, Fe, Nb is largest, indicating that the matrix is a multiferroic PFN phase. On the other hand, in the smaller grains, the content of Co, Zn, Fe, Mn is prominent, which is mainly contributed from CZFMO phase. From the microstructure, the embedment of CZFMO island in the matrix of PFN can be considered as 3-0 type connectivity. Apart from PFN and CZFMO there is no tracing of other phases due to inter diffusion between individual phases as inferred from the XRD data. Here, the synthesis of the composite using a hybrid synthesis technique plays a vital role in the formation of high-quality ceramic composites. Because it is difficult to get dense and homogeneous ceramic composite with solid state reaction route due to the large difference in thermal expansion coefficient and sintering temperature of PFN and CZFMO^15^. During the sintering process, the nucleation and growth process of CZFMO phase continues until all the crystallites begin to impinge on one another. These crystallites are connected to the PFN phase to form aggregates and lead to the formation of voids within the interstices between the aggregates. On further increase in the temperature and concentration of CZFMO, the aggregates developed into grains, and consequently, the grains are distributed homogeneously^15^. Further, the grain distribution is bimodal i.e., PFN matrix having particle size in the < 4 µm range, and CZFMO phase are having particle size < 1.5 µm. The grain size of the PFN and CZFMO phase was calculated from the elemental mapped FESEM image using Image J software considering the larger grain as PFN and smaller grain as CZFMO phase (based on the elemental mapping of FESEM images).The grain size of PFN phase gradually decreases, whereas the grain size of CZFMO increases as shown in Fig. 2f. For the composites, with the increase of CZFMO content up to Φ = 0.3, the smaller grain of CZFMO is gradually trying to evolve against the larger grain of PFN, causing stress in the composite. In the micrograph of Φ = 0.4, the grain growth of CZFMO is more prominent, and the dominance of CZFMO is evident for Φ = 0.4 and 0.5 with nearly equal grain size of the constituent phase which may result in decrease of strain. Hence, Φ = 0.3 can be considered to be an optimum composition, which will be discussed in the subsequent sections.

### Electrical properties

#### Dielectric properties

Figure S4a shows the frequency dependence of dielectric constant (ε_r_) for Φ = 0, 0.1, 0.2, 0.3, 0.4, 0.5 at RT. For all cases, the decreased values of (ε_r_) with increase in frequency representing the polar nature of dielectric materials. The decrease of ε_r_ is prominent in the lower frequency region (i.e., < 10 kHz), and it approaches a constant value at the higher frequency region. The heterogeneity of PFN-CZFMO interface in the composite leads to an increase in the ε_r_ value at the lower frequency region. This can be understood on the basis of Maxwell–Wagner interfacial polarization, as proposed by Koop’s model^29,30^. According to this model, the difference in conductivity of grain and grain boundary leads to increase of dielectric constant. In the composites, the conductivity inhomogeneity between grain and grain boundary results various charge hopping phenomena. i.e., hopping between the respective valence states of cations present in PFN and CZFMO phase. The constant nature of ε_r_ at the higher frequency region is due to the lack of ability of dipoles to respond external applied electric field. The ε_r_ value decreases with the addition of CZFMO phase as the dielectric constant of PFN system is higher than dielectric constant of CZFMO system. However, for the composite Φ = 0.4 and 0.5, the ε_r_ value is higher as compared to the other values of Φ (= 0.1, 0.2, and 0.3) at lower frequency region. This could be attributed to existence of excess space charge carriers formed at the interface between PFN grains, which is surrounded by excess CZFMO grains. So, Φ = 0.3 can be considered as the percolation limit of CZFMO phase. The tangent loss (tanδ) also decreases with increase in frequency and increases with the addition of CZFMO content (shown in Fig. S4b). From Fig. S4b, it can be observed that tanδ increases significantly in the lower frequency region with the addition of CZFMO content (Φ = 0.1, 0.2, 0.3), and it appears to be merged in the higher frequency region. However, the tanδ value increases with an increase in CZFMO content (Φ = 0.4, 0.5) throughout the investigated frequency range. The increase of tanδ with an increase of CZFMO content may be due to the increase in the conductivity as a result of itinerant electrons at the interface between PFN and CZFMO phase. In Fig. S4c, the comparison of ε_r_ and tanδ with composition at RT is shown. The value of tanδ is < 0.1 up to Φ = 0.3 and after that it reaches to 0.6. This quick increase of dielectric loss after Φ = 0.3 refers to the percolation limit of the composite. Above the percolation threshold, the sudden increase of dielectric loss is due to the formation of conduction path and leakage current from the conducting ferrite phase^31^.

#### Piezoelectric properties

Figure S4d shows the variation of piezoelectric coefficient (d_33_) with Φ. The piezo-electric coefficients were measured after the electrical poling of the samples at 20 kV/cm. The d_33_ value of PFN is 107 pC/N, and with the addition of CZFMO, it decreases to 19 pC/N (Φ = 0.5). The decrease of d_33_ value with increase concentration of CZFMO phase could be attributed to the decrease in resistance and volume of PFN content in the composite. This is because the resistance of CZFMO phase is smaller as compared to PFN phase. The composite with a higher content of CZFMO cannot sustain higher voltage due to low resistance, leads to a decrease in the piezoelectric properties^32^. The behaviour of d_33_ follows the same trend as that of dielectric constant.

#### Ferroelectric properties

**Figure 3:**
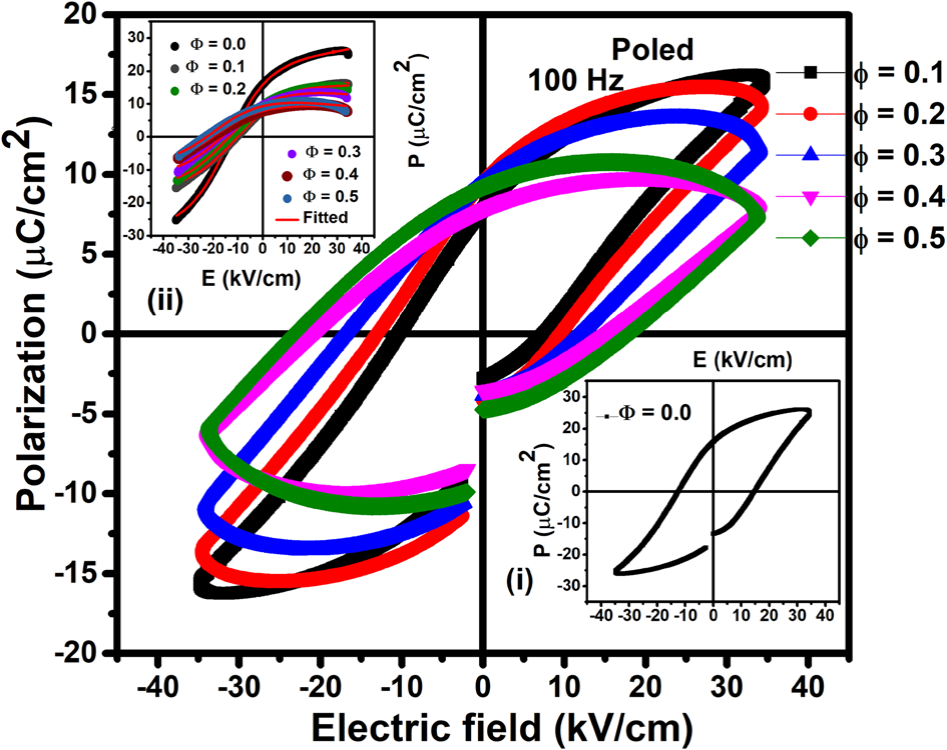
Variation of electrical polarization (P) with electric field (E) measured at RT for PFN-CZFMO composites. Inset (i) P-E loop for Φ = 0.0 and (ii) fitting of the upper branch of P-E loop.

To examine the existence of ferroelectricity in these composites, we have measured the polarization (P) versus electric field (E) of the composites at RT and is shown in Fig. 3. We have performed P-E hysteresis measurements with and without poling and the results were compared as shown in the Fig. S5 for Ф = 0.2 and 0.5 as representative. In the case of E-poling better P-E hysteresis was observed. In case of multiferroic composites the presence of free carriers and defects cannot be avoided. During poling, the free charge carrier may get sufficient energy to move from one place to another and that may compensate the deficiencies in the composite. Consequently, better shape of P-E hysteresis loop is expected. Similar type of observation has been reported by our group^33–35^. Figure 3, shows the P-E hysteresis of the composites after electrical poling. Inset of Fig. 3 (i) is the RT P-E loop of PFN, showing well-saturated hysteresis nature confirming the presence of ferroelectric behaviour. The composites also exhibit a typical hysteresis loop confirming the existence of ferroelectric properties. However, the composites Φ = 0.1, 0.2 and 0.3 show a better saturated hysteresis loop as compared to Φ = 0.4, 0.5. The slightly unsaturated P-E loop for Φ = 0.4, 0.5 is due to the leaky nature of CZFMO phase. The voltage drops across CZFMO phase due to the conducting nature puts a constraint on domain switching, resulting in slightly unsaturated P-E loop^36^.

In order to separate the intrinsic and extrinsic (due to the conductivity) polarization contributions, the P-E loops have been fitted. For saturated dipolar polarization hysteresis curves, the dipole switching dependence on electric field can be written as $P_{d} (E) = - P_{d}^{ + } ( - E)$, where the (+) sign is for the branch of polarization is positive going field ramp and (−) sign stands for negative going field ramp and

2 $$ P_{d}^{ + } (E) = P_{S} \tanh \left[\frac{{E - E_{C} }}{2\delta }\right],\;\;\delta = E_{C} \left[ {\log \left( {\frac{{1 + P_{r} /P_{S} }}{{1 - P_{r} /P_{s} }}} \right)} \right]^{ - 1} $$

where $P_{d}^{ + } (E)$ is the dipolar polarization, *P*_*S*_ is the saturated polarization, *P*_*r*_ is the remnant polarization and *E*_*C*_ is the coercive field^37^. In case of a lossy/leaky dielectric system, the polarization (P_loss_) is not an intrinsic effect, rather it is coming from conductivity, which is related to charge *Q* = *σEAt*, where *σ* = conductivity, *E* = electric field, *A* = cross-sectional area and *t* = time^38^. So, the obtained P-E loops can be fitted by combining both intrinsic (dipolar) and conductivity contribution. During fitting of the P-E loops, the lower branch could not be fitted accurately due to discontinuity along the polarization axis at zero applied fields. This type of behaviour generally depends upon the way of measurement carried out and the capacity of the material to retain the polarization charge when the biased field is turned off. During the measurement of P-E curve, by the application of one triangular wave cycle, the program will preset the starting polarization value *−P*_*r*_. The second hysteresis loop can be obtained by applying another triangular wave cycle and the delay between these two wave cycles leaves the capacitor at 0 V for 1 s. The loss of polarization value in this time delay gives a gap in the P-E loops. Also, the origin of discontinuity in the P-E loops may be due to internal field generated due to space charges^39^. The polarization loss in case of ferroelectric and multiferroic materials is due to the leakage current through the capacitor. The gap width increases with the increase in the increase in the conductivity (charge loss) of the capacitor. From the Fig. 3, it has been observed that the gap in case of the composite is more as compared to the pure PFN.

We have fitted the PE loop only for the upper branch by considering both the intrinsic and conductivity contributions to the polarization. Inset of Fig. 3ii shows the fitting of the upper branch of P-E loop and a reasonably good fit is observed between the experimental and simulated data. The different ferroelectric parameters obtained after fitting the experimental data is tabulated Table 2. It has been observed that the P_S_ values decrease whereas E_C_ values increase with an increase in CZFMO phase. The decrease of the P_S_ values or the increase in the P_loss_ value may be due to the presence of large amount of non-ferroelectric CZFMO phase. The obtained value of P_loss_ is very small as compared to the Ps (due to the dipolar polarization). So the contributions of P_loss_ due to the conductivity is very small for lower phase fraction of CZFMO. However for higher concentrations this effect is clearly visible. This non-ferroelectric phase also obstructs the domain wall motion of the ferroelectric region and increases E_C_^33^. The increase of Ec with an increase of magnetic material concentration is due to the pinning of ferroelectric domains by CZFMO (magnetic content) having higher conductivity.

**Table 2:** Compositional dependence saturation polarization (P_S_), remanent polarization (P_r_), Co-ercive field (E_C_) and P_loss_ obtained after fitting of the P-E loop at RT for the composites.

Composition (Φ)	P_s_ (µC/cm^2^)	P_r_ (µC/cm^2^)	E_C_ (kV/cm)	P_loss_ (µC/cm^2^)
0.0	22.36	15.77	13.72	0.004
0.1	17.51	5.75	7.63	0.018
0.2	15.99	9.40	13.45	0.143
0.3	13.32	7.25	14.01	0.756
0.4	10.27	7.91	19.15	1.052
0.5	9.54	6.99	18.37	1.750

### Magnetic properties

**Figure 4:**
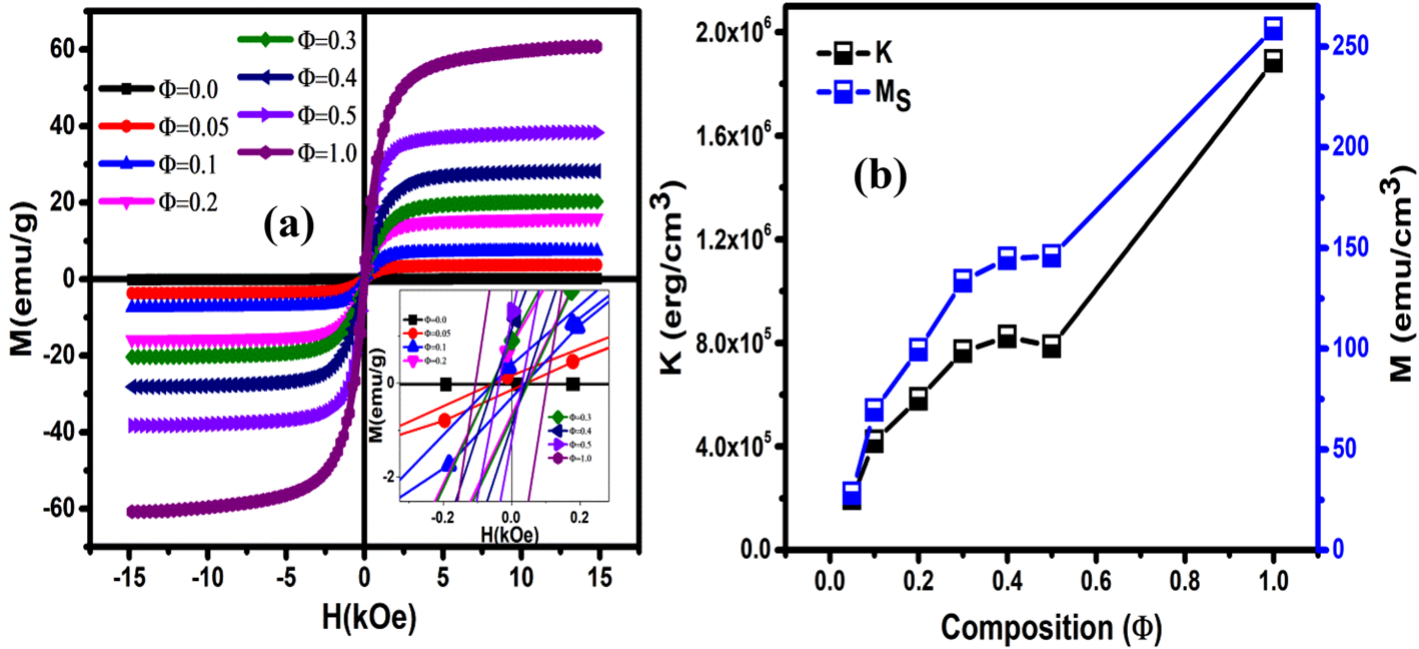
(**a**) The variations of magnetization (M) with magnetic field (H) at RT for all the compositions. Inset shows the enlarged view of the graph. (**b**) Variation of anisotropy constant (K) and saturation magnetization (M_S_) with composition obtained from LAS method.

The magnetic field dependence of magnetization for PFN-CZFMO recorded at RT is shown in Fig. 4a. For all these composites, the loop is complete, thin with a small opening of the hysteresis loop, which specifies soft magnetic behaviour of the material. The M-H curves are not saturated for the applied magnetic field of 15 kOe for all the composition^40^. So, the saturation magnetization can be calculated by using the law of approach to saturation (LAS) method^40^. According to this method, the magnetization obtained at highest field can be expressed as

3 $$ M = M_{S} [1 - \beta K^{2} /M_{S}^{2} H^{2} ] $$

The terms M_S_, H, K represent saturation magnetization, applied external magnetic field, cubic anisotropy constant respectively. Here β = 8/105 for random polycrystalline material with cubic anisotropy^41^. The graph of M versus 1/H^2^ is plotted in the high magnetic field region H ≥ H_C_ (i.e., near the saturation). For the fitting, magnetization data above H = 10 kOe (greater than H_C_) are used, where the intercept gives the value of M_S_ and the anisotropy constant can be calculated from the slope using Eq. (3). The obtained values of M_S_ and K are plotted for all values of Φ in Fig. 4b. An increase in M_S_ and K with the addition of CZFMO content has been observed. The prepared ceramic composites PFN-CZFMO are the mixture of multiferroic PFN phase and ferrite CZFMO phase. So, the magnetic hysteresis is coming due to the interaction between the antiferromagnetic behaviour (weak FM) of PFN and ferrimagnetic behaviour of CZFMO phase. It has been reported that PFN exhibits weak FM behaviour due to spin clustering and canted ordering of spins even for temperatures above T_N_^20^. Moreover the coupling of two order parameters allows the multiferroic materials to exhibit magnetism (above magnetic transition temperature of FM/AFM) inside the domain wall ordering, as long as it is in the ferroelectric phase^42^. However, the magnetic behaviour of CZFMO dominance over the magnetic property of PFN and mostly the magnetic contribution is from CZFMO phase. Therefore, in order to extract the FM and AFM/PM contribution of the constituent phases, we have tried to fit the experimentally obtained M-H data via the equation given below: ^43^.

4 $$M(H) = \left[ {\frac{{2M_{FM}^{S} }}{\pi }\tan^{ - 1} \left\{ {\left( {\frac{{H \pm H_{ci} }}{{H_{ci} }}} \right)\tan \left. {\frac{{\pi M_{FM}^{R} }}{{2M_{FM}^{S} }}} \right\}} \right.} \right] + \chi H$$

where the first term is for FM contribution and the second liner part (χ*H*) is arising due to AFM/PM contribution. The expressions M, M^S^_FM_, H_ci_, M^R^_FM_ and χ represent measured magnetization in the presence of applied magnetic field, saturation magnetization (weak FM), co-ercivety and magnetic susceptibility (AFM and/or PM) respectively. This equation gives a reasonably good fit between the experimental and theoretical model for all the compositions, which are shown in Figure S6 (supplementary material) for Φ = 0.1, 0.2, 0.3, 0.5 as representatives. All the magnetic parameters M_S_, M_r_, H_C_ and $\chi$ obtained after fitting are mentioned in Table 3. The increased in M^S^_FM_ values have been observed with increase of CZFMO concentration. The magnetic co-ercivity values increases from Φ = 0.1 to 0.3, and after that, it reduces. As H_C_ is inversely related to particle size^44^, the decrease of H_ci_ after Φ = 0.3 composition may be due to the increase in particle size of the CZFMO phase as obtained from FESEM study. Also, the decrease of H_ci_ after Φ = 0.3 might be due to the percolation limit of the composites. A similar type of result is observed for BCT-NCZF composites^45^. The remnant magnetization and magnetic susceptibility values increase with an increase in Φ. This increase in M_S_ and M_r_ are well expected because it mainly depends on the ferrite component CZFMO in the composites.

**Table 3:** The magnetic parameters (obtained from the fitting of M-H loop) and obtained values of $\frac{\Delta \alpha }{d},$ H_bias_, 2β/d and 3γ/d parameters for the composites (obtained from magnetoelectric coupling measurements).

Composition (Φ)	M_S_ (emu/g)	M_r_ (emu/g)	H_C_(Oe)	*χ*	$\frac{\Delta \alpha }{d}$(mV /cm-Oe)	H_bias_ (Oe)	2β/d (mV/cm-Oe^2^)	3γ/d (mV/cm-Oe^3^)
0.05	3.90	0.12	33.72	8.29 × 10^–6^	4.96	3200	0.0025	− 3.5 × 10^–7^
0.1	7.76	0.27	34.40	1.62 × 10^–5^	6.00	2404	0.0059	− 1.7 × 10^–7^
0.2	17.06	0.52	35.44	4.95 × 10^–5^	15.43	2303	0.0072	− 1.6 × 10^–7^
0.3	22.47	0.55	36.18	9.17 × 10^–5^	26.78	2303	0.0159	− 3.7 × 10^–7^
0.4	30.95	0.66	30.44	1.17 × 10^–4^	21.21	2702	0.0125	− 3.2 × 10^–7^
0.5	40.66	1.14	25.98	1.15 × 10^–4^	8.14	2304	0.0073	− 1.7 × 10^–7^
1.0	62.10	4.73	96.27	1.49 × 10^–4^				

### Magneto-dielectric properties

**Figure 5:**
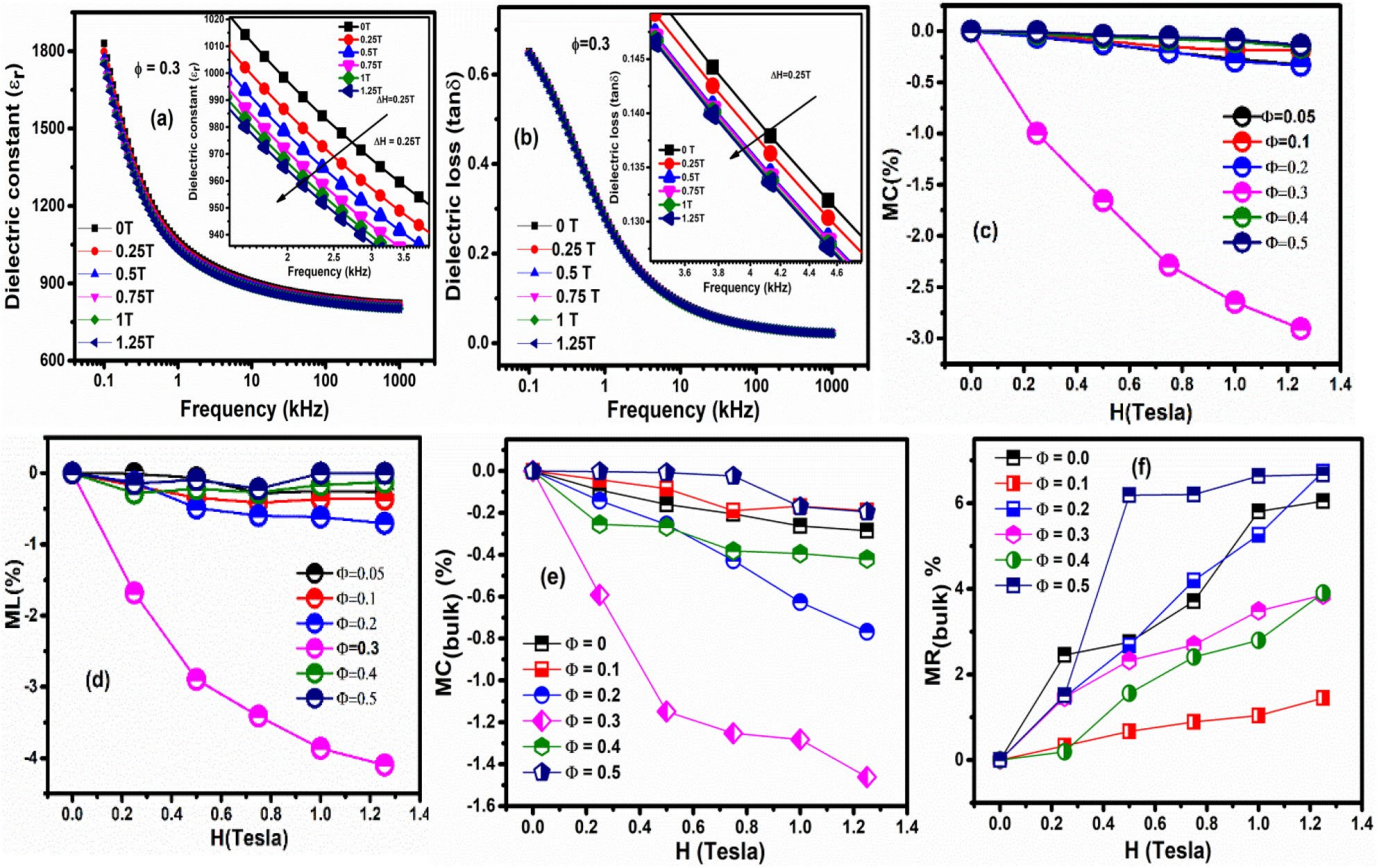
Variation of (**a**) dielectric constant (ε_r_) and (**b**) dielectric loss (tanδ) versus frequency for Φ = 0.3 in presence of various magnetic fields respectively. Inset shows the enlarged view of the graph to show the effect of magnetic field to these dielectric parameters. Variation of calculated (**c**) magneto-capacitance (MC%) and (**d**) magneto-loss (ML%) with applied magnetic fields for various compositions. Variation of (**e**) MC_(bulk)_% and (**f**) MR_(bulk)_% with magnetic field obtained from the fitting of the Nyquist plot.

The ME coupling permits to tune electrical polarization (P) by the application of magnetic field (H) or to change magnetization (M) with the help of the electric field (E). It is well established that ME coupling behaviour can be investigated from magneto-dielectric measurement^5^. The true nature of ME coupling in a material can be confirmed from the two criteria: 1st one is the appearance of anomaly around the magnetic transition temperature while performing temperature-dependent dielectric measurement. The 2nd one is the changes in dielectric permittivity by the application of external magnetic field^5^. In order to examine RT magneto-dielectric effect in PFN-CZFMO composites, frequency dependent dielectric measurement was carried out at various magnetic fields at RT. Figure 5a,b shows the variation of ε_r_, tanδ with frequency at various magnetic fields for the composites Φ = 0.3 (considering as the representative of composites), respectively. It can be noticed that with the increase in applied magnetic fields (inset of Fig. 5a) the ε_r_ values decreases. This type of behaviour suggests the presence of negative ME coupling in the materials. The negative value of ME coupling behaviour depends on the spin pair correlations of the neighbouring spins and the coupling constant^5^. The tanδ also shows similar nature as that of frequency dependent ε_r_, i.e., tanδ decreases by the application of magnetic fields. This change in dielectric parameters i.e. tangent loss and dielectric constant in the presence of magnetic fields satisfied the second criteria and indicated the presence of magneto-electric coupling in the materials. Thus the presence of magneto-dielectric effect can be quantified by calculating magneto-capacitance (MC(%)) and magneto-loss (ML(%)). The MC(%), ML(%) values are calculated by using the formula as reported elsewhere^5^. The dependence of MC(%) and ML(%) with the magnetic field for all the composition is shown in Fig. 5c,d. The MC(%) and ML(%) tending towards more negative values with an increase in magnetic fields. With the increase in weight percentage of CZFMO phase, both MC(%) and ML(%) increases in negative values up to Φ = 0.3, after this composition again, these value decreases. Thus a maximum value of MD behaviour is observed for Φ = 0.3, compared to all other compositions.

In case of poly crystalline materials, magnetoelectric/magneto-dielectric properties may not be entirely from the bulk (i.e., gain) and hence contribution form interfaces (grain boundary and material-electrode) with different resistivity cannot be ignored^46^. The dielectric properties significantly affected by the space charge effect due to the above interface layers^46^. In case of multiferroic systems, the work function difference between the electrode material and multiferroic can affect the dielectric properties when device under test is placed in a magnetic field, which is not a true magneto-dielectric behavior^47^. The presence of different extrinsic (interfacial) effect in poly crystalline can be separated from the bulk contribution using Magneto-impedance measurement. So, we have plotted the complex impedance plot (−Z″ vs. Z′ i.e., Nyquist plot) for various static magnetic field for composition Φ = 0.2 and 0.5 (a representative case is shown in Figure S7). Similar behaviours have been observed for all other compositions. The experimental data has been fitted using the equivalent circuit by commercially available software ZSimp Win Version 3.21 for all composites at different magnetic field. For lower compositions (< Φ = 0.3), the presence of single semicircular arc in the impedance data has been fitted using the equivalent circuit (RCQ), where (RCQ) signifies parallel combination of bulk resistance (R_b_), bulk capacitance (C_b_) and CPE (Q = constant phase element (CPE)). For higher compositions, the overlapping semicircular arc of the impedance data has been fitted using the equivalent circuit (RCQ)(RC). The (RCQ)(RC) represent the parallel combination of bulk resistance (R_b_), bulk capacitance (C_b_) and CPE along with another parallel combination of grain boundary resistance (R_gb_), grain boundary capacitance (C_gb_) connected in series. The justification of choosing of the equivalent circuits is described in our previous work^47–49^. A good fit between the experimental and theoretical data has been observed, suggesting the appropriate choice of equivalent circuit for the fitting. The bulk magneto-capacitance (MC_bulk_(%)) and bulk magneto-resistance (MR_bulk_(%)) are calculated (form bulk resistance and bulk capacitance obtained after fitting the magneto-impedance data using the proposed equivalent circuit) and plotted as shown in Fig. 5e,f. It has been observed that the bulk magneto-capacitance decreases with the increase in the applied magnetic field, where as the bulk magneto-resistance increases with increase in the applied magnetic field. So the changes in the bulk magneto-capacitance with applied magnetic field suggesting that the observed magneto-dielectric behavior is also contributed form magneto-capacitance, which are intrinsic in nature.

In order to establish the nature of ME coupling in PFN-CZFMO composites, we have performed magneto-dielectric measurements at different magnetic fields 0 ≤ H ≤ 12.5kOe as a function of frequency at a step of 2.5kOe magnetic field. The nature of ME coupling can be probed from the Landau free energy equation used for single-phase multiferroic system. The Landau Free energy equation is given by^47,50^:

5 $$G = \frac{1}{{2\varepsilon_{0} }}P^{2} - PE - \alpha PM + \beta P^{2} M + \delta P^{{}} M^{2} + \gamma P^{2} M^{2} .....$$

where P is the spontaneous polarization, E is the external electric field, M is the spontaneous magnetization, ε_0_ is the dielectric susceptibility, α, β, δ, γ are ME coupling coefficients. Here α is the linear ME coefficient and β, δ, γ are higher order ME coefficients. The nature of ME coupling i.e., liner (PM) coupling, quadratic linear (P^2^M), and biquadratic coupling (P^2^M^2^) can be identified by solving the above expression. Out of these, the quadratic linear coupling is forbidden from the symmetry point of view^47,50,51^. Hence, there will be two possibilities i.e., either linear or biquadratic.

**Figure 6:**
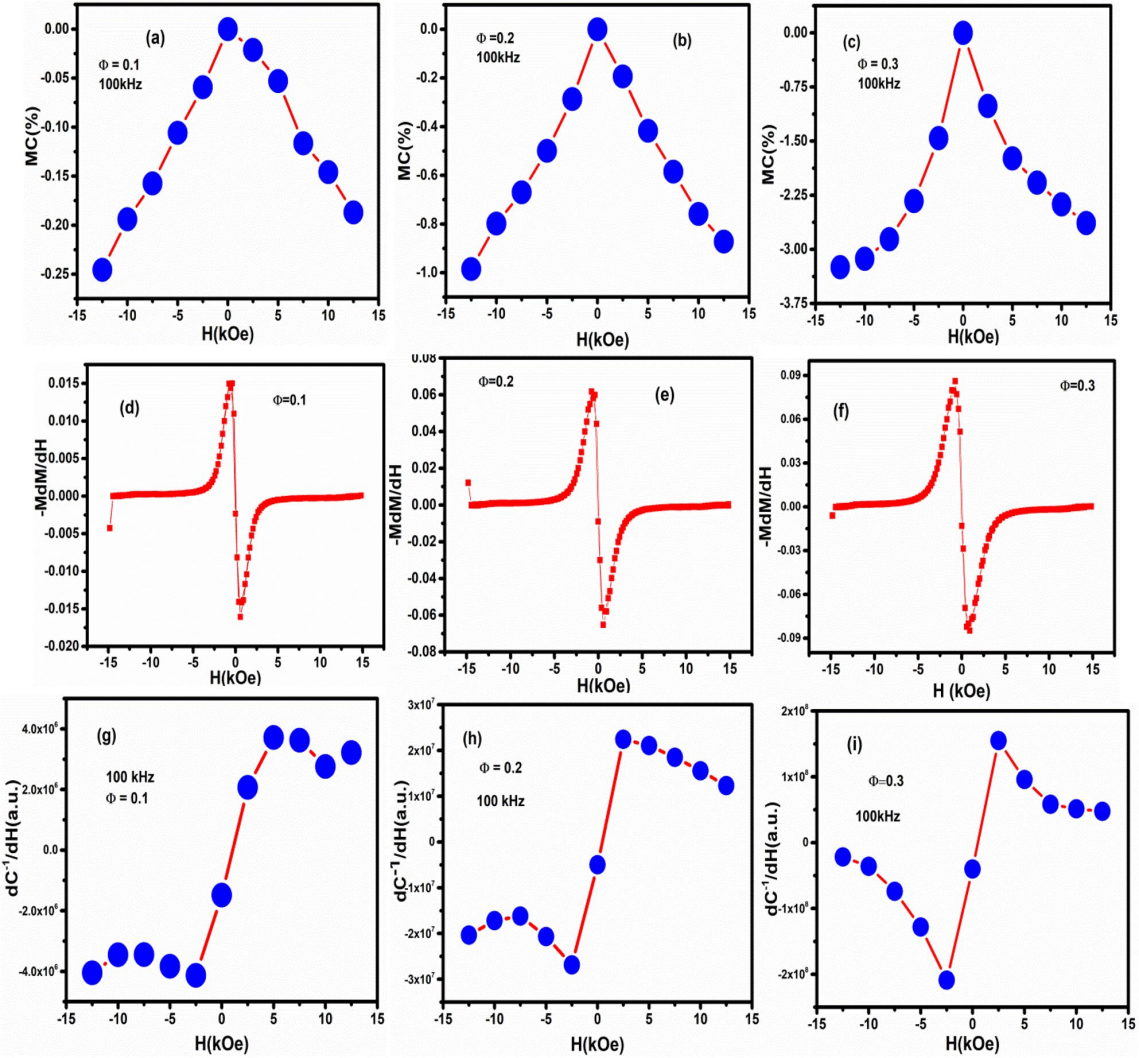
(**a**–**c**) Variation of MC% in both +Ve and –Ve applied magnetic fields for Φ = 0.1, 0.2, 0.3 of (1 − Φ) PbFe_0.5_Nb_0.5_O_3_-Φ Co_0.6_Zn_0.4_Fe_1.7_Mn_0.3_O_4_ respectively at 100 kHz. (**d**–**f**) variation of –M × (dM/dH) with magnetic fields for Φ = 0.1, 0.2, 0.3 respectively. (**g**–**i**) variation of dC^−1^/dH with magnetic fields for Φ = 0.1, 0.2, 0.3 respectively.

Under equilibrium condition $\frac{dG}{{dP}} = 0$, the expression for inverse of electrical susceptibility $\chi^{ - 1} = \frac{{d^{2} G}}{{dP^{2} }}$. As C^−1^ (inverse of capacitance) is the directly measurable quantity and it can be scaled as χ^−1^. By taking the first-order derivative of C^−1^ with the magnetic field and relating with the magnetic field, the nature of ME can be probed. Now, if the ME coupling is linear (PM) then dχ^−1^/dH response should be zero for all applied magnetic fields, However, if there is quadratic linear (P^2^M) coupling then dχ^−1^/dH is proportional to dM/dH. In case of biquadratic (P^2^M^2^) coupling behaviour dχ^−1^/dH must be proportional to M(dM/dH)^47,50^. So, to probe the nature of coupling, we have plotted the experimentally obtained quantity and verified the nature of coupling. The variation of MC%, -M × (dM/dH) and dC^−1^/dH with magnetic field (H) are shown in Fig. 6 for Φ = 0.1, 0.2 and 0.3 as representative. Similar behaviour has been observed for other samples. The capacitance (measured) values from high frequency (100 kHz) data were taken to avoid space charge polarization. It can be observed from the graph that the behaviour of dC^−1^/dH is strongly similar to the properties of − M × (dM/dH). Hence there is an existence of biquadratic coupling in PFN-CZFMO composites. The biquadratic coupling can be explained in terms of the magnetostrictive and piezoelectric effect that results into square of the respective order parameter P^2^M^2^. So, the application of magnetic field induces a magnetostriction in the magnetic phase and produces strain in the system. The induced strain transmits to the piezoelectric phase and via piezoelectric effect the strain modifies the electrical order parameters (i.e., polarization), hence the electrical properties^47^. Here, α = δP/δH is the product of the piezomagnetic deformation δs/δH and the piezoelectric charge generation δQ/δs^50^.

### Magneto-electric coupling

The above study on the P-E, and M-H reveal the simultaneous presence of ferroelectric and ferromagnetic behaviour in the composite at RT. The existence of MD behaviour also suggests the coupling between the order parameters. This gives rise to ME coupling in the composite. There are two characteristics modes such as active and passive modes are used for the measurement of direct ME effect^52^. The active mode of ME measurement is similar to that of measurement of MD effect where a current is allowed to pass through the sample, and change in capacitance is measured by the application of the magnetic field. In the active mode, a direct current-pulse voltage is applied to the sample, and change in polarization signal induced by the magnetic field is obtained (P-E hysteresis loop). During the measurement, there is also a contribution from the magnetoresistance and Maxwell–Wagner interfacial polarization effect. In the passive mode, instead of the application of test current and voltage, the induction of ME voltage is measured through an applied magnetic field. Consequently, the contribution from magnetoresistance and Maxwell–Wagner interfacial polarization effect is excluded. However, measuring in dynamically induced voltage by the application of small alternating current magnetic field superimposed on a magnetic field bias, much attention is given to distinguish the real ME effect and Faraday effect^4^.

**Figure 7:**
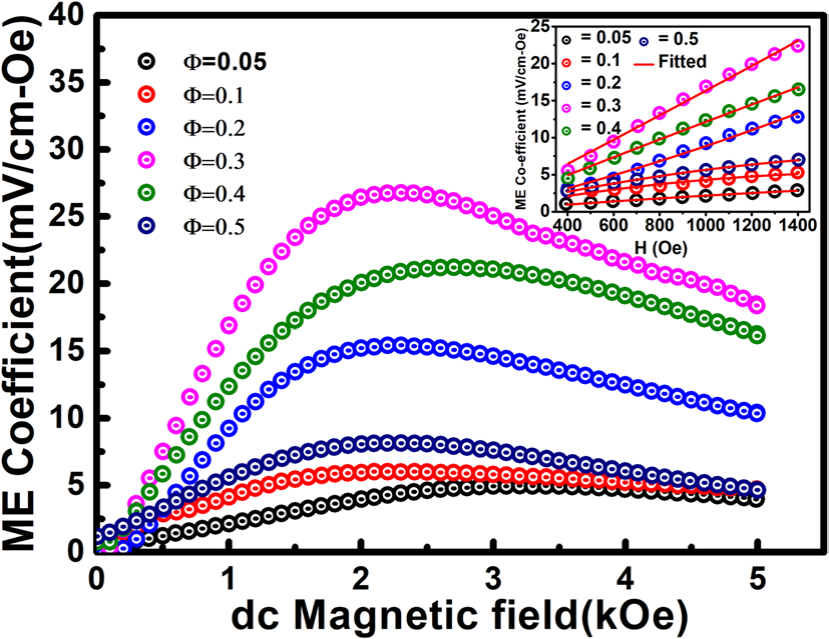
Variation of ME coefficient with applied dc magnetic field for (1 − Φ) PFN-Φ CZFMO composites recorded at RT. Inset shows the fitting of the ME coefficient vs. dc magnetic field below H_C_ using Eq. (6).

The change in the ME coefficient ($\frac{\Delta \alpha }{d}=\frac{(\alpha \left(H, {h}_{0}\right)-\alpha (0, {h}_{0})}{d})$ vs. dc magnetic field (H) with an ac magnetic field of frequency 1.008 kHz and ac magnetizing field of 1 Oe for the composites are shown in Fig. 7. In Fig. 7, it is observed that the ME coefficient rises gradually with an increase in the magnetic field, reaches a maximum, and with further increase of the magnetic field, it decreases gradually for all the composites. This type of behaviour is generally related to the magnetic field dependence of magnetostriction^24^. The increasing characteristics of ME coefficient in the range of 2.3 kOe–3.2 kOe is due to the growth of domains with the applied magnetic field; above this field, the further growth of domain decrease the deformation caused in the decreasing value of ME coefficient. However, the deformation can still increase while the ME coefficient can decrease. This effect happened because the ME effect is proportional to the derivative of the deformation^53–55^. It is well known that magnetostriction gets saturated above the critical magnetic field for known magnetic materials. The parent magnetic compound CoFe_2_O_4_ shows critical saturation in magnetostriction nearly 4 to 5 kOe applied external magnetic field, which shifted towards lower magnetic field side with an increase in Zn and Mn substitution^56,57^. Hence the present composites show maximum ME coupling coefficients near the critical magnetostriction field, which is about 2.3 kOe magnetic field in the present case for Ф = 0.3. In other words, the increase of ME coefficient up to a particular dc bias magnetic field is due to the optimum value of magnetostriction with the applied field.

The induced ME coefficient can be expressed by using the following equation^58^.

6 $$ \frac{dE}{{dH}} = \frac{V}{{h_{0} d}} = \frac{{\alpha + 2\beta H + 3\gamma H^{2} + 4\delta H^{3} }}{d} $$

where E represent the electric field, H is magnetic field, h_0_ is the magnitude of the ac magnetic field, d is the thickness of the material, α is the linear components of ME coefficient, β, γ, δ are higher order nonlinear ME coefficients. In order to find out the contributions of higher order non-linear ME coefficients terms, we have fitted the change in the ME coefficient ($\frac{\Delta \alpha }{d}$) vs. magnetic field (H) below H_C_ by considering the above equation up to 3rd term (and neglecting the higher order terms) as shown in inset of Fig. 7. The fitting parameter i.e., (2β/d) obtained after fitting the experimental data is listed in Table 3. From the Table 3 it has been observed that (2β/d) is found to be 0.0025 mV/cm-Oe^2^ for Ф = 0.0.5 and it increases with the increase in the CZFMO concentrations. The maximum value is found to be 0.0159 mV/cm-Oe^2^ for Ф = 0.3. Above this concentration it’s value decreases. This 2nd order term i.e., (2β/d) represents the interfacial effect of both PFN and CZFMO phases. There is one-to-one correspondence between the variation of micro-strain and (2β/d) with the change in CZFMO concentrations. So the magneto electric coefficient is the contributions of interfacial phenomena mediated through the strain, but not due to the bulk phenomena^58^. The value of (3γ/d) is found to be negative and the value is very small (~ × 10^−7^ mV/cm-Oe^3^). However, physical origin behind the negative value of 3γ/d is needs to be investigated.

**Figure 8:**
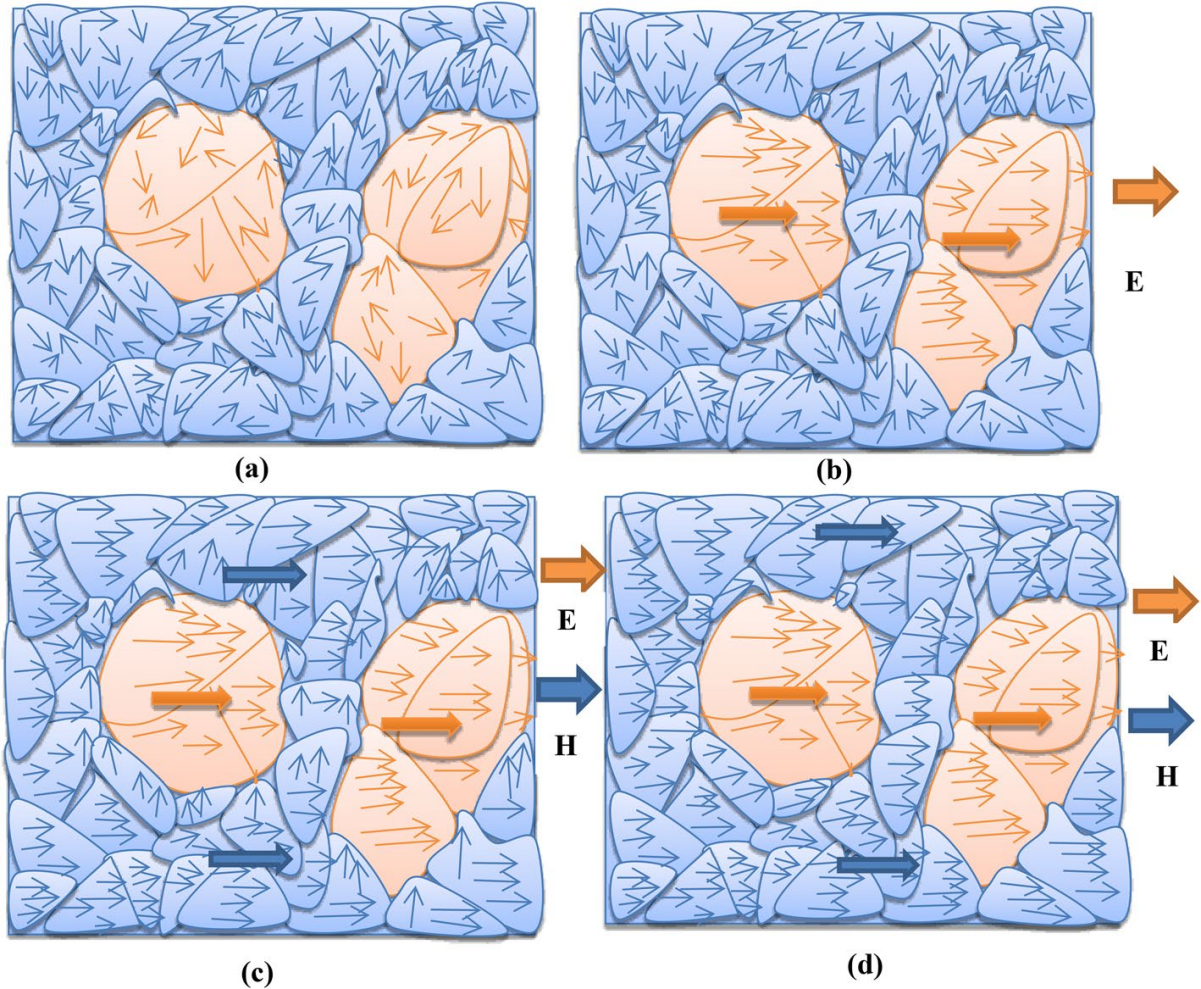
Schematic representations of evoluation of magnetic (blue colour) and electric (orange colour) domains for different magnetic bias.

The mechanism behind the strain mediated coupling at the interface between the constituent phases of the composite could be explained using the phase field modelling and simulations as reported by Ma et al.^59^ In order to explain the ME behaviour, we have drawn the magnetostrictive-ferroelectric phase morphology (i.e., grain microstructure) of the particulate composite similar to the FESEM micrograph for Ф = 0.3 as shown in the Fig. 8a. The 0–3 particulate composite is expected to exhibit the isotropic properties. The ferroelectric (piezoelectric) and magnetic (magnetostrictive) phases are characterized by the domain level process and the domain behaviours are very sensitive to the interface of the phases and the grain boundary of the composite^60–62^. In case of as prepared particulate composite (i.e., the ferroelectric and magnetic phases are unpoled), the local polarization and magnetization vectors are expected to be randomly oriented (with few vortices) as shown in Fig. 8a. By the application of electric field (20 kV/cm) in order to pole the ferroelectric phase/domain in the composite, the local polarization vectors are oriented along the direction of the applied electric field (i.e., the poling direction)^63^. Even after the removal of the electric field, the remnant polarization (P_r_) will be retained by the ferroelectric phase (equilibrium state). The total macroscopic polarization is represented by a big arrow as shown in Fig. 8b. The finite conductivity of the magnetic phase also helps in effective poling and assisting the poled state of the ferroelectric phase^59^. For the Ф = 0.3 composite we have observed small but finite conductivity, so we are expecting the saturation polarization can be achieved after poling. Now the saturation polarization (P_S_) is not zero, but M_S_ = 0, i.e., magnetic dipoles are randomly oriented as shown in Fig. 8b. By the application of bias magnetic field for measuring the ME voltage coefficient, the local magnetization vectors of the magnetostrictive phase are aligned along the direction of the magnetic field, which generate strain^62^. However, the magnetization vectors near the interface of both the phases will not be parallel to the applied magnetic field for lower applied bias magnetic field as shown in Fig. 8c. With further increase in the magnetic field, the magnetization vectors will be rotated towards the magnetic field direction. Due to the rotation of magnetic vectors at the interface, the magnetostrictive strain can be more effectively transferred elastically across the interface with the poled ferroelectric phase for inducing polarization. Such elastic interaction is strong near the interface. So the magneto-electric coupling of the composite is an indirect coupling (as predicted in the previous section as biquadratic nature) of the domain level, which is mediated through strain at the interface. The value of the magneto-electric coupling is the efficiency of the transferring the strain of the magnetostrictive phase to an electrostrictive phase through the elastic interaction between them. The decrease of ME coefficient beyond the critical field (Fig. 7) could be attributed to the saturation response (all magnetic vectors are parallel to the applied magnetic field direction) of the ferrite phase to the further application of the magnetic field as shown in Fig. 8d^59,63^.

The maxima of ME coefficient ($\frac{\Delta \alpha }{d}$) obtained to be around 26.78 mV/cm-Oe for Φ = 0.3 under H_bias_ = 2303 Oe and then decreases to 8.14 mV/cm-Oe for Φ = 0.5 under H_bias_ = 2304 Oe. The magnitude of optimum H_bias_ decreases from 3200 to 2702 Oe with an increase in CZFMO content. The variation of maximum $\frac{\Delta \alpha }{d}$ and H_bias_ for different CZFMO concentrations is represented in Table 3.The magnitude of maximum $\frac{\Delta \alpha }{d}$ = 26.78 mV/cm-Oe for Φ = 0.3 is found to be larger as compared to different ferroelectric/multiferroic-ferrite composites, which is compared in Table 4. It has been reported that stronger ME effect in the composite depends on the magnetostriction coefficient, magnetization of the magnetostrictive phase and piezoelectric coefficient of the piezoelectric phase. Also the phase connectivity of the ferrite phase in the composite in such a way that the accumulated charge should not leak through the magnetostrictive phase and also have good poling strength of the composites. In the prepared composite PFN-CZFMO, PFN has piezoelectric coefficient of 107 pC/N, CZFMO has good magnetostriction coefficient^22^ with high resistivity and the prepared composite is poled by the application of electric field of 20 kV/cm. Here, PFN can also exhibit ferromagnetic characteristics at RT or above due to spin clustering even if after T_N_ (145 K)^20^. Among these composites, Φ = 0.3 shows highest ME coefficient with M_S_ = 22.47 emu/g, d_33_ = 48 pC/N. So we have observed the maximum ME coefficient for composite Φ = 0.3 due to the maximum micros-strain, optimized value of magnetization, magnetostrictions and piezoelectric coefficients. For Φ = 0.4, 0.5, in spite of higher magnetic properties, the overall resistivity of the PFN-CZFMO composite decreases and also leaking of the charge carrier takes place due to the increase in the itinerants electrons at the interface between the constituent phases. Consequently, leaking of the charge carrier obstruct electric poling and cause difficulty for the rotation of dipole by the application of magnetic field. This leads to decrease in ME coupling coefficient in the composite^24,64^. The ferrite/ferroelectric based composite with higher resistivity, lower Hc and reduced value of magnetic anisotropy can show improved ME coefficient that can be operated by applying lower magnetic fields.

**Table 4:** Comparison of ME coupling coefficient of (1 − Φ) PFN-Φ CZFMO (Φ = 0.3) with other composite materials.

Materials	ME coefficient	References
(0.7) PFN-0.3 CZFMO	26.78 mV/cm-Oe	This work
(1 − *x*) ((0.94)(Na_0.5_Bi_0.5_)TiO_3_)-0.06(BaTiO_3_))–*x* Co_0.6_Zn_0.4_Fe_1.7_Mn_0.3_O_4_	8.2 mV/cm-Oe	^24^
(1 − *x*) Pb_0.93_La_0.07_(Zr_0.60_Ti_0.40_)O_3_-NiFe_2_O_4_	8.9 mV/cm-Oe	^65^
Pb(Zr_0.52_Ti_0.48_)O_3_-CoFe_2_O_4_	21 mV/cm-Oe	^66^
BiFeO_3_-CoF_2_O_4_	16 mV/cm-Oe	^67^
K_0.5_Na_0.5_)NbO_3_-LiSbO_3_-CoFe_2_O_4_	15.01 mV/cm-Oe	^68^
(1 − *x*) (Ni_0.8_Zn_0.1_Cu_0.1_)Fe_2_O_4_-*x* [0.48Pb(Ni_1/3_Nb_2/3_)O_3_–0.02Pb(Zn_1/3_Nb_2/3_)O_3_–0.05Pb(Ni_1/2_W_1/2_)O_3_–0.45PbTiO_3_]	7.34 mV/cm-Oe	^69^
(1 − *x*)[0.948K_0.5_Na_0.5_NbO_3_-0.052LiSbO_3_]-*x*Ni_0.8_Zn_0.2_Fe_2_O_4_	20.14 mV/cm-Oe	^64^
NiFe_2_O_4_-(Ba_0.8_Sr_0.2_)TiO_3_	0.43 mV/cm-Oe	^70^
BaTiO_3_-(Li,Co)Fe_2_O_4_	0.375 mV/cm-Oe	^71^
(Na_0.5_Bi_0.5_)TiO_3_)-NiFe_2_O_4_	0.155 mV/cm-Oe	^72^

## Conclusions

Pore-free multiferroic PFN-CZFMO particulate composites were successfully synthesized using a hybrid synthesis technique. The structural study suggested the formation of composites and induction of lattice strain due to lattice mismatch between PFN and CZFMO phase. The P-E and M-H study show existence of both FE and FM behaviour indicating the multiferroic nature of the composites at RT. The MD measurement carried out at RT reveals the existence of ME coupling behaviour, and the highest MC% is observed for Φ = 0.3. The composite Φ = 0.3 having the optimum value of M_S_ = 22.47 emu/g, d_33_ = 48 pC/N showing highest ME coefficient of 26.78 mV/cm-Oe applicable in ME devices. The variation dχ^−1^/dH (or dC^−1^/dH) with H is similar to the H dependence of M(dM/dH), which is in accordance with Landau’s free energy expression and hence suggesting biquadratic (P^2^M^2^) nature of magneto-electric coupling. The complex domain level strain mediated coupling mechanism between the magnetic and ferroelectric phase is responsible for the appearance of ME coupling in the particulate composite. These composites might be suitable for next-generation low power memory, magnetic field sensors and other multi-functional devices.

## Supplementary Information

Supplementary Information.
