# Supplementary material for: Unravelling the nature of magneto-electric coupling in room temperature multiferroic particulate (PbFe0.5Nb0.5O3)–(Co0.6Zn0.4Fe1.7Mn0.3O4) composites
Source: Sci Rep. 2021 Feb 4;11:3149. doi: 10.1038/s41598-021-82399-7 (PMC7862596; doi:10.1038/s41598-021-82399-7)
Supplement: Supplementary file 1 — Supplementary Information. [file 41598_2021_82399_MOESM1_ESM.pdf]

Unravelling the Nature of Magneto-electric Coupling in Room Temperature Multiferroic Particulate ($PbFe_{0.5}Nb_{0.5}O_3$) - ($Co_{0.6}Zn_{0.4}Fe_{1.7}Mn_{0.3}O_4$) Composites

Krishnamayee Bhoi[1], H. S. Mohanty[1], Ravikant[2], Md. F. Abdullah[1], Dhiren K. Pradhan[3], S. Narendra Babu[4], A. K. Singh[1], P. N. Vishwakarma[1], A. Kumar[2], R. Thomas[5,6], Dillip K. Pradhan[1*]

[1] Department of Physics and Astronomy, National Institute of Technology, Rourkela, Odisha-769008, India

[2] CSIR National Physical Laboratory, Dr. K. S. Krishnan Marg, New Delhi-110012, India

[3] Extreme Materials Initiative, Geophysical Laboratory, Carnegie Institution for Science, Washington, DC 20015, USA

[4] Materials Research Laboratory, Department of Physics, Osmania University, Hyderabad-500007, India

[5] Division of Research and Development, [6]School of Chemical Engineering and Physical Sciences, Lovely Professional University, Jalandhar-Delhi G.T. Road, Phagwara, Punjab– 144411, India

Figure Captions (Supplementary Material)

Figure. S1 W-H plots of (a) PFN (b) CZFMO phase of 0.7PFN-0.3CZFMO composite.

Figure. S2 (a) TEM image (b) HRTEM image (c) SAED pattern of CZFMO. Figure S2 (d) HAADF-STEM with corresponding elemental mapping of Co, Zn, Fe, Mn, O elements in CZFMO.

Figure. S3 (a) FESEM micrograph and (b) EDS spectra of PFN.

Figure. S4 Variation of (a) dielectric constant (ε_r) (b) dielectric loss (tanδ) with frequency for different value of Φ, (i.e., Φ = 0.0, 0.1, .2, 0.3, 0.4, 0.5). Compositional dependence of (c) ε_r and tanδ at 10 kHz and (d) piezoelectric constant (d_{33}) at RT.

Figure. S5 Effect of poling on the P-E loop of the composites for (i) Φ = 0.2 and (ii) Φ = 0.4.

Figure. S6 Fitting of the M-H data for (1-Φ) $PbFe_{0.5}Nb_{0.5}O_3$-$\Phi Co_{0.6}Zn_{0.4}Fe_{1.7}Mn_{0.3}O_4$ (Φ= 0.1 (a), 0.2 (b), 0.3 (c), 0.5(d)) recorded at RT. Inset shows the magnified view for the low magnetic field region.

Figure. S7 Complex impedance (Nyquist) plots for Φ = 0.2 (a), 0.5 (b) composites for different applied magnetic field. Inset shows the equivalent circuit to fit the impedance data.

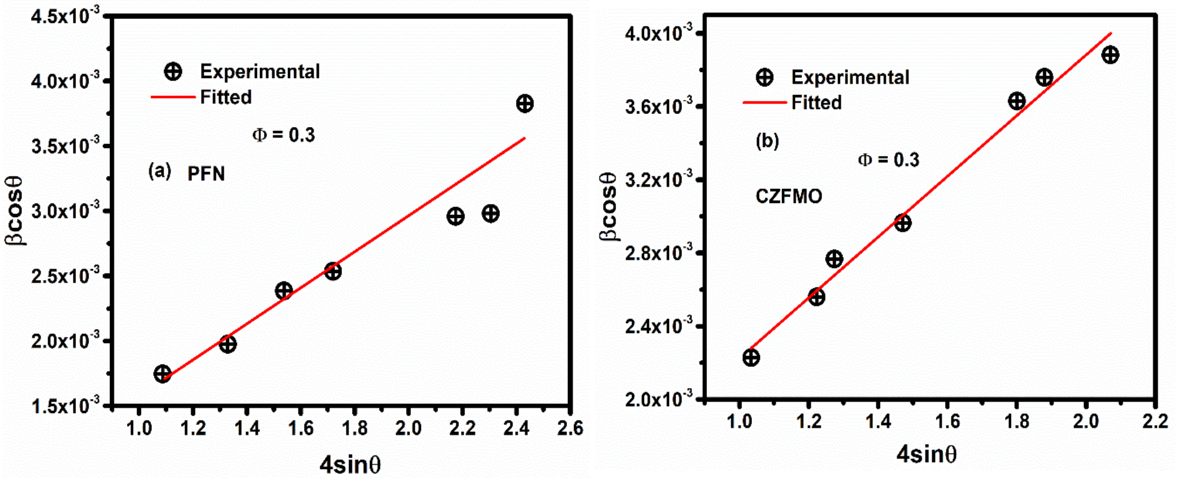

Figure. S1 W-H plots of (a) PFN (b) CZFMO phase of 0.7PFN-0.3CZFMO composite.

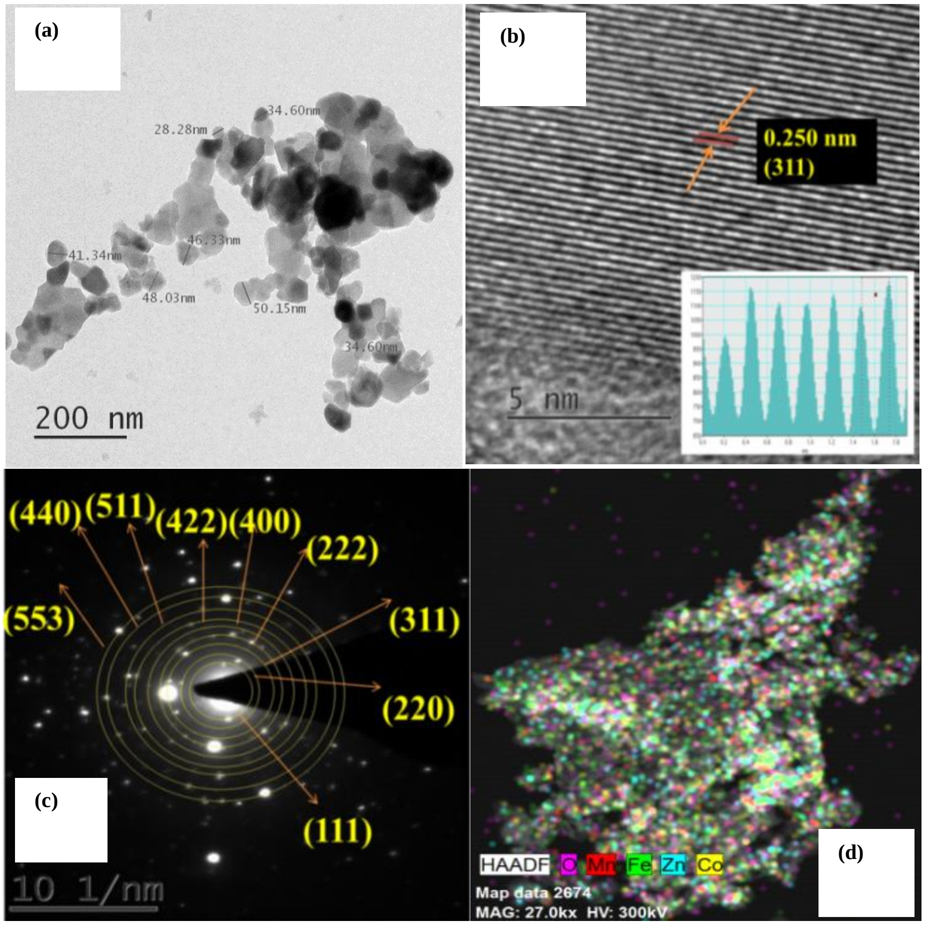

Figure. S2 (a) TEM image (b) HRTEM image (c) SAED pattern of CZFMO. Figure S2 (d) HAADF-STEM with corresponding elemental mapping of Co, Zn, Fe, Mn, O elements in CZFMO.

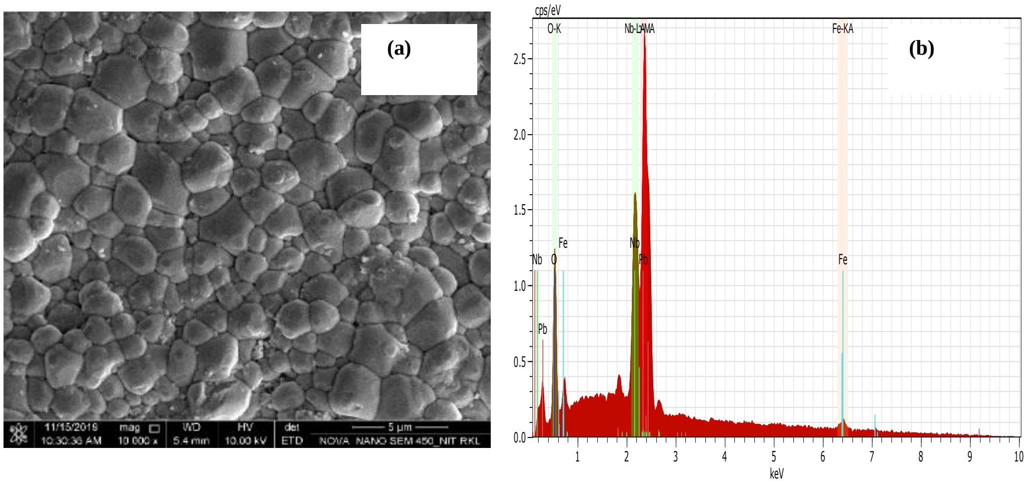

Figure. S3 (a) FESEM micrograph and (b) EDS spectra of PFN.

.

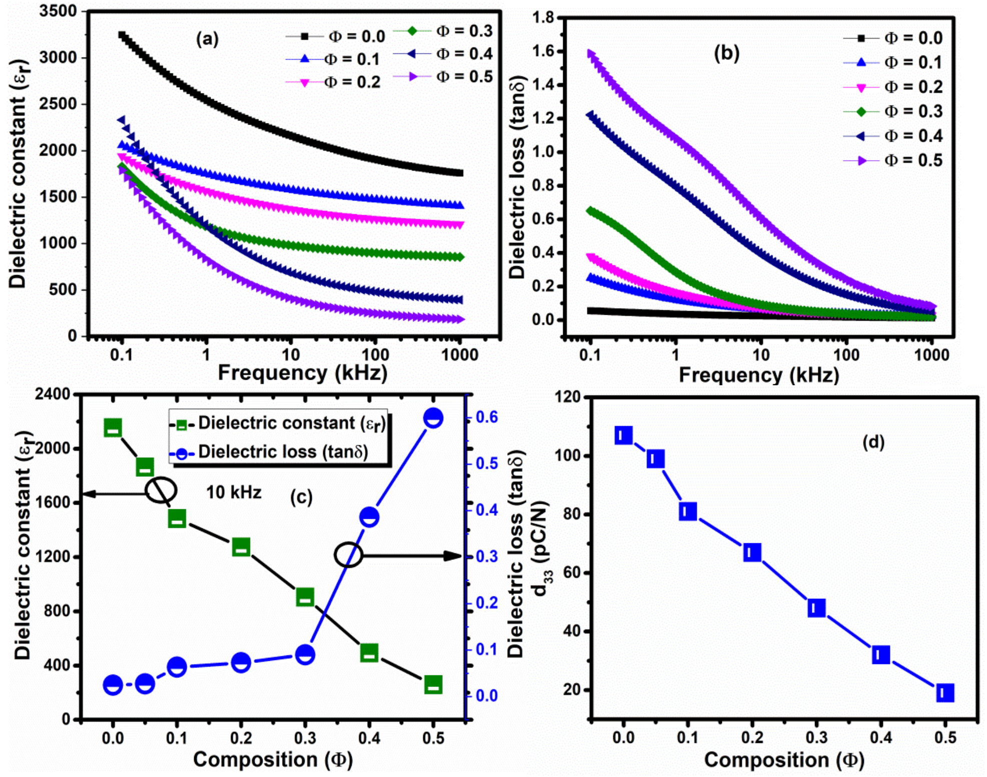

Figure. S4 Variation of (a) dielectric constant (ε_r) (b) dielectric loss (tanδ) with frequency for different value of Φ, (i.e., Φ = 0.0, 0.1, .2, 0.3, 0.4, 0.5). Compositional dependence of (c) ε_r and tanδ at 10 kHz and (d) piezoelectric constant (d_{33}) at RT.

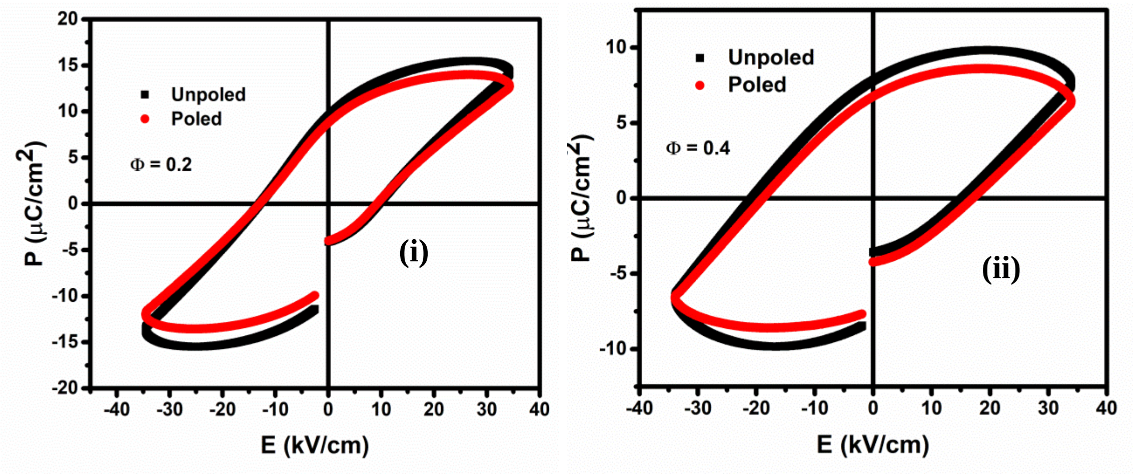

Figure. S5 Effect of poling on the P-E loop of the composites for (i) Φ = 0.2 and (ii) Φ = 0.4.

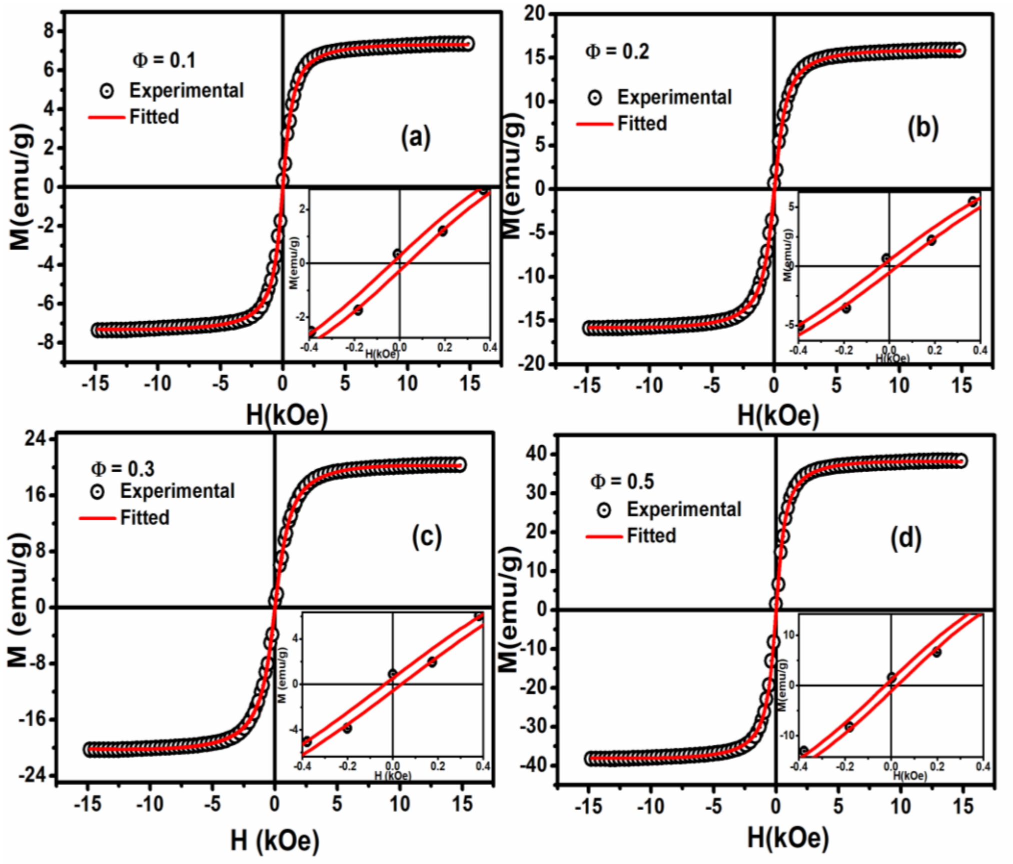

Figure. S6 Fitting of the M-H data for (1-Φ) $PbFe_{0.5}Nb_{0.5}O_3$-Φ$Co_{0.6}Zn_{0.4}Fe_{1.7}Mn_{0.3}O_4$ (Φ= 0.1 (a), 0.2 (b), 0.3 (c), 0.5(d)) recorded at RT. Inset shows the magnified view for the low magnetic field region.

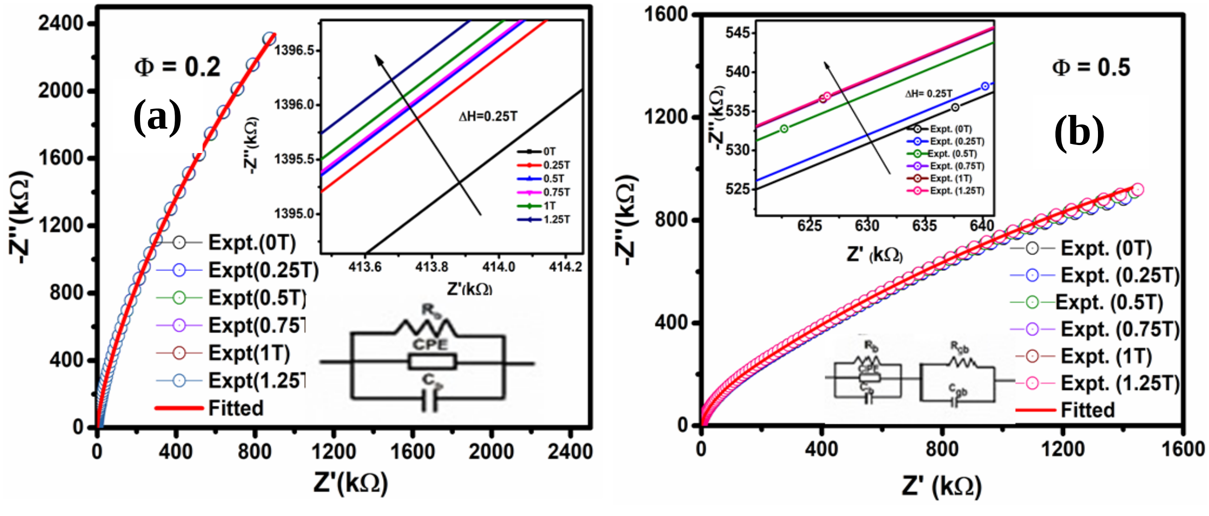

Figure. S7 Complex impedance (Nyquist) plots for Φ = 0.2 (a), 0.5 (b) composites for different applied magnetic field. Inset shows the equivalent circuit to fit the impedance data.
